# Pathophysiological mechanisms of bradycardia in patients with anorexia nervosa

**DOI:** 10.1002/hsr2.331

**Published:** 2021-07-23

**Authors:** Reiner Buchhorn, Christoph Baumann, Christian Willaschek

**Affiliations:** ^1^ Department of Pediatrics Caritas‐Krankenhaus Bad Mergentheim Bad Mergentheim Germany; ^2^ Medical Faculty University of Wuerzburg Würzburg Germany

**Keywords:** adolescent, anorexia nervosa, autonomic nervous system, electrocardiography, heart rate

## Abstract

**Background:**

The purpose of this investigation was to examine heart rate variability (HRV), interbeat interval (IBI), and their interrelationship in healthy controls, bradycardic hyperpolarization‐activated cyclic nucleotide‐gated channel 4 (*HCN4*) mutation carriers, and patients with anorexia nervosa (AN). We tested the hypothesis that neural mechanisms cause bradycardia in patients with AN. Therefore, we assumed that saturation of the HRV/IBI relationship as a consequence of sustained parasympathetic control of the sinus node is exclusively detectable in patients with AN.

**Methods:**

Patients with AN between the ages of 12 and 16 years admitted to our hospital due to malnutrition were grouped and included in the present investigation (N = 20). A matched‐pair group with healthy children and adolescents was created. Groups were matched for age and sex. A 24‐hour Holter electrocardiography (ECG) was performed in controls and patients. More specifically, all patients underwent two 24‐hour Holter ECG examinations (admission; refeeding treatment). Additionally, the IBI was recorded during the night in *HCN4* mutation carriers (N = 4). HRV parameters were analyzed in 5‐minute sequences during the night and plotted against mean corresponding IBI length. HRV, IBI, and their interrelationship were examined using Spearman's rank correlation analyses, Mann‐Whitney *U* tests, and Wilcoxon signed‐rank tests.

**Results:**

The relationship between IBI and HRV showed signs of saturation in patients with AN. Furthermore, signs of HRV saturation were present in two *HCN4* mutation carriers. In contrast, signs of HRV saturation were not present in controls.

**Conclusions:**

The existence of HRV saturation does not support the existence of parasympathetically mediated bradycardia. Nonneural mechanisms, such as *HCN4* downregulation, may be responsible for bradycardia and HRV saturation in patients with AN.

## 1 INTRODUCTION

Anorexia nervosa (AN) has the highest mortality rate among psychiatric disorders.^1^, ^2^ Other than suicide, cardiovascular and respiratory complications are the leading causes of mortality.^3^ Typically, these deaths are unexpected and attributed to cardiac arrhythmia.^4^ The causes of cardiac arrhythmia and their overall impact on cardiovascular function remain under investigation. Bradycardia, which is commonly observed in patients with AN, likely plays a crucial role in the pathophysiology of AN.^4^

To provide insights into the neurophysiology of AN and etiology of bradycardia, heart rate variability (HRV) has been examined in patients with AN in many investigations.^5^, ^6^ HRV measures the variations in R‐R interval, which reflect a complex interplay of feedback loops, thermogenesis, intrinsic mechanisms of pacemaker cells, and parasympathetic and sympathetic tone. Abnormal HRV has been associated with numerous cardiovascular and non‐cardiovascular pathologies.^7^, ^8^, ^9^, ^10^, ^11^ In a large number of research articles, high HRV parameters are associated with a high vagal tone.^6^, ^12^, ^13^, ^14^, ^15^ Conversely, low HRV is often associated with sympathetic dominance.^7^, ^9^, ^11^, ^14^, ^15^

In patients with AN, mostly high HRV parameters were observed during the acute phase of the disease. It was therefore believed that bradycardia in patients with AN is mediated via a high cardiac vagal tone and high parasympathetic influence.^4^, ^5^, ^6^

Interestingly, in the last few years, it has been suggested that HRV is partly a nonlinear surrogate for heart rate (HR).^16^, ^17^, ^18^, ^19^ Studies conducted by D'Souza et al imply that alterations in intrinsic HR via the regulation of ionic channels are primarily responsible for HRV and bradycardia in athletes.^18^, ^19^ Consequently, the theory of increased vagal tone or decreased sympathetic tone as a cause of starvation‐ or exercise‐induced bradycardia has been questioned by some researchers.^16^, ^17^, ^18^, ^19^, ^20^, ^21^ Likewise, HRV metrics as an index of autonomic regulation have been challenged.^16^, ^22^

## 2 METHODS

Considering these arguments, we analyzed HRV and interbeat interval (IBI) length in nighttime electrocardiography (ECG) segments of patients with AN before and during refeeding therapy, patients with hyperpolarization‐activated cyclic nucleotide‐gated channel 4 (*HCN4*) mutations, and healthy controls. HRV metrics, their dynamics during refeeding therapy, and their relationship with IBI may offer new insights into the underlying mechanisms of bradycardia in patients with AN.

**FIGURE 1:**
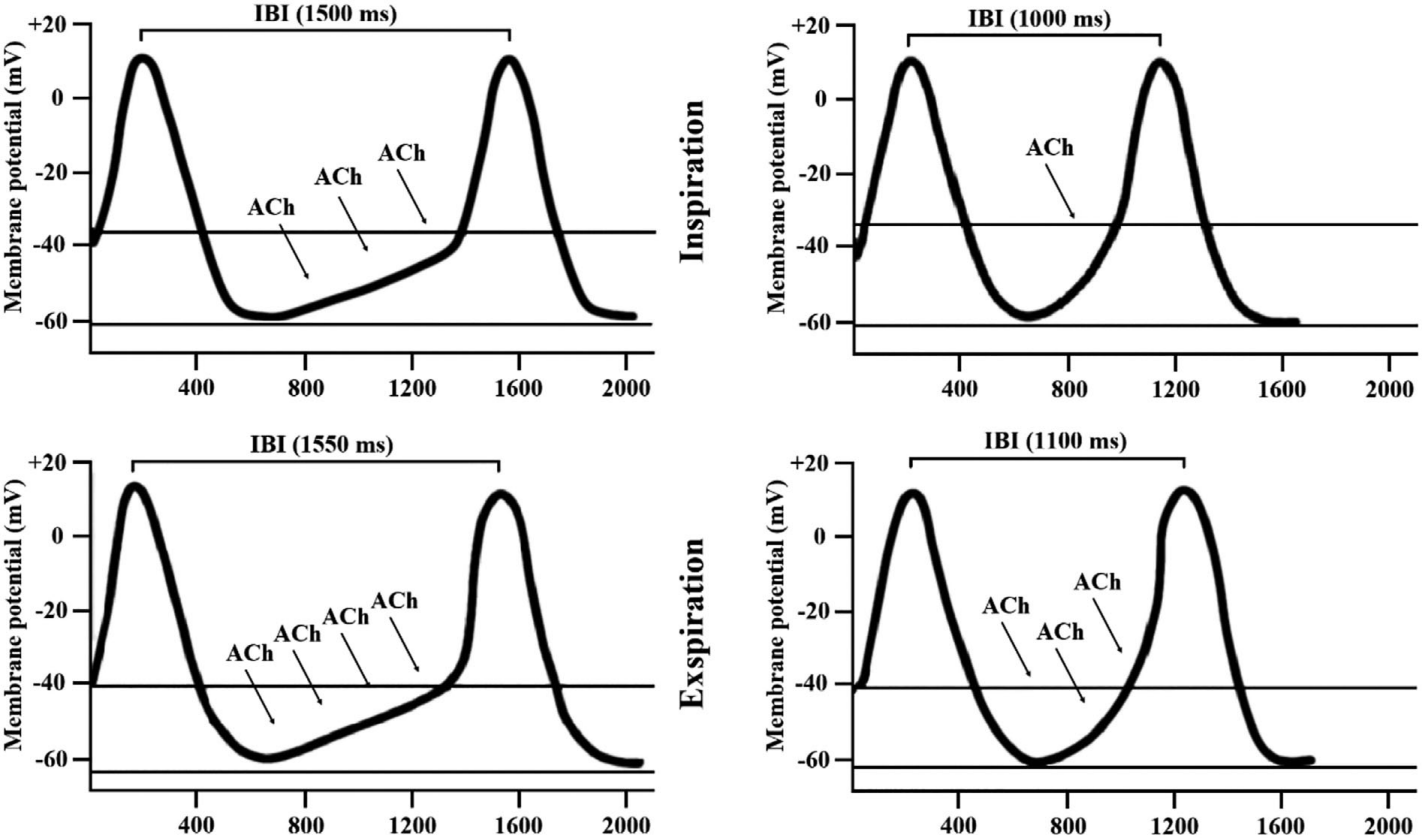
Heart rate variability saturation effect. Respiration‐related episodic acetylcholine (ACh) release (heart rate variability [HRV] saturation on the left side, no HRV saturation on the right side). During each breathing cycle, parasympathetic‐mediated variation in heart rate (HR) naturally occurs. During inspiration, activated baroreceptors diminish vagal tone via the central autonomic network, which is followed by an increase in HR. In contrast, during expiration, baroreceptors are less active and HR decreases.^30^ In patients with anorexia nervosa, these fluctuations in the breathing cycle may attenuate at low HRs during certain sleep stages with a high parasympathetic influence. During expiration, the vagus nerve releases high amounts of ACh, which cannot be sufficiently cleaved by low acetylcholinesterase levels.^45^, ^46^ As a result, the expected decline in the concentration of ACh during inspiration in the sinus node area may not occur, and high concentrations of ACh persist during inspiration and expiration (left side). This sequence of events leads to sustained parasympathetic control of the sinus node during the whole breathing cycle, which eliminates respiratory cardiac modulation and reduces HRV at very low HRs.^8^ From: de Geus; Should heart rate variability be “corrected” for heart rate? Biological, quantitative, and interpretive considerations; CC BY 4.0; www.onlinelibrary.wiley.com/doi/full/10.1111/psyp.13287

While nonneural mechanisms of bradycardia, such as *HCN4* downregulation or *HCN4* c.1737+1G>T mutation, presumably express themselves as a boost in HRV, drug‐induced parasympathetic dominance leads to a saturation effect in HRV metrics.^23^, ^24^, ^25^ This association shows that HRV may constantly increase with a decline in HR in *HCN4* mutation carriers or subjects with HCN4 downregulation. At the same time, HRV uncouples from changes in HR at a certain point and then decreases with longer IBIs in subjects with a high vagal tone. The underlying mechanism of HRV saturation is probably high‐intensity vagal nerve discharge during expiration as part of respiratory sinus arrhythmia. If sufficient acetylcholine (ACh) is released during expiration, ACh concentrations in the sinus node area remain high during inspiration, and the parasympathetic effect persists during the whole respiratory cycle, resulting in low HRV and HR (Figure 1).^23^, ^24^ Thus, our hypothesis suggests that HRV saturation occurs in patients with AN, whereas such an effect remains unverifiable in controls and patients with the *HCN4* c.1737+1G>T mutation, given that bradycardia in patients with AN is caused by increased cardiovagal modulation and not by nonneural mechanisms, such as HCN4 downregulation. In this investigation, we discuss the pathophysiological mechanisms of bradycardia and reveal the changes in HRV and HR during in‐hospital refeeding treatment.

### 2.1 Patients

#### 2.1.1 Patients with AN and controls

Twenty patients who met the Diagnostic and Statistical Manual of Mental Disorders V criteria for AN were admitted to our hospital between June 2015 and December 2018 and included in the present analysis.^26^ Patients with severe depression, anxiety disorders, obsessive‐compulsive disorders, psychotic disorders, sleep disorders, developmental disorders, and cardiovascular diseases in their medical history were excluded. For all patients, parents consented to a 24‐hour ECG. Additionally, all patients were supervised by a psychologist.

The treatment protocol was standardized. Physicians prescribed diets starting around 1200 kcal per day and then increased by at least 200 kcal/day up to a normal caloric intake of 2500 to 3000 kcal. Targeted caloric intake was achieved by supplementing most patients with high‐energy liquid nutrition, starting with 10 kcal/kg/day. Nasogastric tube feeding was not required. Patients were supervised during meals and snacks for up to 30 minutes post‐consumption by patient sitters, primarily nurses. Patients were weighed each morning on the same scale under standardized conditions (ie, consistent clothing and weighing before visiting the bathroom). Fluid balance, blood count, and serum electrolytes were measured at admission and later, according to the doctor's orders. Nutritional supplements were routinely administered (ie, omega‐3‐fatty acids, calcium glycerol phosphate, zinc gluconate, magnesium, B vitamins, vitamin C, and vitamin K). Controlled exercise phases were allowed. Outdoor walking or running, gymnastics, and physiotherapy were offered to patients. Following sufficient weight gain and clinical stabilization, all patients were referred to a psychotherapeutic clinic specializing in eating disorders or supervised in an outpatient setting.

#### 2.1.2 Patients with *HCN4* mutations

Three female patients and one male patient carrying the *HCN4* c.1737+1G>T mutation who consented to nighttime IBI recordings were included (age: 13, 23, 27, and 22 years, respectively). Prof. Robert Sepp recruited these patients from a former study.^27^ Patients did not take any drugs during IBI recording, and they were not athletes.

### 2.2 Ethics statement

All procedures were performed in accordance with the ethical standards of the appropriate ethics review board and with the 1964 Helsinki declaration and its later amendments. All study participants and their parents provided written informed consent for data acquisition and analysis. The study design was approved by the university ethics committee (University of Würzburg, study number 143/16‐mk).

Data acquisition from patients with *HCN4* c.1737+1G>T mutation was performed by Prof. Robert Sepp, who recruited these patients from a former study. Molecular genetic analyses and ECG recordings in these patients were approved by the Institutional Ethics Committee of University of Szeged, Hungary. For the control group, we used a database containing 151 24‐hour ECGs from healthy individuals aged 2 to 23 years.^28^ The database was composed of German and Polish children and adolescents. For Polish children and adolescents, data analysis and acquisition were approved by Poznan University of Medical Science Bioethical Committee.

### 2.3 Heart rate variability

For an exhaustive explanation of all standard HRV metrics, their calculation, meaning, and clinical use, we referred to studies by Shaffer et al, Hayano et al, and the guidelines of the Task Force of the European Society of Cardiology and the North American Society of Pacing and Electrophysiology.^15^, ^29^, ^30^, ^31^

### 2.4 Design

We examined the effects of nutritional refeeding on HRV parameters and HR in 20 adolescents with AN during in‐hospital refeeding. To assess changes in HRV parameters, a 24‐hour ECG was performed at admission and during nutritional refeeding. The second ECG was recorded between fifth and 14th days post‐admission when a high‐calorie intake (>1500 kcal) and patient compliance were achieved. For the control group, we used a database containing 151 24‐hour ECGs from healthy individuals aged 2 to 23 years.^28^

Each patient was randomly matched to a control subject by age (±1 year) and sex. The monitoring period started between 07:00 and 12:00 and was discontinued on the second day. The Medilog Darwin ECG system (Holter recorder: Medilog FD4 or Medilog AR4, Schiller, Switzerland; sampling rate 250 Hz) was used for all ECG recordings of controls and patients with AN. An experienced cardiologist edited and inspected all ECG recordings for non‐sinus rhythm or artifacts. Notepad++ software was used to count beat annotations. ECGs with <95% sinus beats were excluded. For *HCN4* mutation carriers, we used Polar H10 chest belts to record nighttime RR intervals in an ambulant setting. Further, 24‐hour ECGs were used to rule out severe dysrhythmia in these patients.

Time‐domain (td) and frequency domain (fd) parameters were calculated from exported 5‐minute IBI segments using Kubios HRV Premium 3.1.0 software for all patients with AN, controls, and patients with *HCN4* c.1737+1G>T mutations.^32^ Non‐sinus beats and artifacts were corrected using an automatic correction algorithm. The following td and fd parameters were determined: root means square of successive differences (RMSSD), percentage of successive NN intervals that differed by more than 50 ms (pNN50), and natural logarithm of spectral power high frequency (LnHF, 0.15‐0.40 Hz) power in absolute units. LnHF power was calculated based on the Lomb‐Scargle periodogram. A smoothing factor of 0.02 Hz was applied.

### 2.5 Statistics

For statistical analysis, we selected 5‐minute sequences of recordings in sleeping patients and controls at night. The nighttime sequences were identified by the time designation of our recordings (22:00‐06:00). For the analysis, we excluded segments that showed >5% software‐corrected artifacts according to Kubios software. All other segments recorded during sleep were included in the analysis. The fluctuations in HR and autonomic tone during different sleep stages were desired and enabled evaluating the IBI/HRV relationship in patients and controls. HRV and mean IBI were calculated for each patient with AN in 90 sequences of 5 minutes each at admission and 85 sequences of 5 minutes each during refeeding treatment on an average. In the controls, HRV and mean IBI were calculated in 87 sequences of 5 minutes each on an average, whereas for patients with *HCN4* c.1737+1G>T mutation, HRV and IBI were calculated in 86 sequences of 5 minutes each on an average. Median nighttime HRV values for each patient and control subject were calculated. Based on the median values, mean values were calculated for each group (Table 1).

**TABLE 1:** Patient characteristics and HRV metrics data are shown as mean ± SD

		Age (years)	Disease duration (months)	Calorie intake (kcal)	Mean night‐time IBI (ms)	RMSSD (ms)	pNN50 (%)	LnHF
Healthy controls		14.00 ± 1.26	‐	‐	885.98 ± 136.54†‡	55.86 ± 23.84†‡	28.01 ± 18.26†‡	6.78 ± 0.97†‡
Patients with *HCN4* mutations		21.25 ± 5.91	‐	‐	1218.09 ± 230.93	124.14 ± 25.83	62.03 ± 8.39	8.46 ± 0.52
Patients with anorexia nervosa	At admission	14.00 ± 1.34	8.92 ± 6.70	829.09 ± 416.30	1391.15 ± 283.94†#	113.63 ± 48.28†	60.86 ± 12.87†	8.39 ± 0.82†
	During re‐feeding	‐	‐	2641.18 ± 416.30	1075.91 ± 165.41‡#	107.90 ± 45.27‡	54.65 ± 17.06‡	8.20 ± 0.89‡

*Note*: Significant differences: controls versus patients with AN at admission ‐> †(*P* < 0.01); controls versus patients with AN at release ‐> ‡(*P* < .01); patients with AN at admission versus patients with AN at release ‐> §(*P* < .01).

**FIGURE 2:**
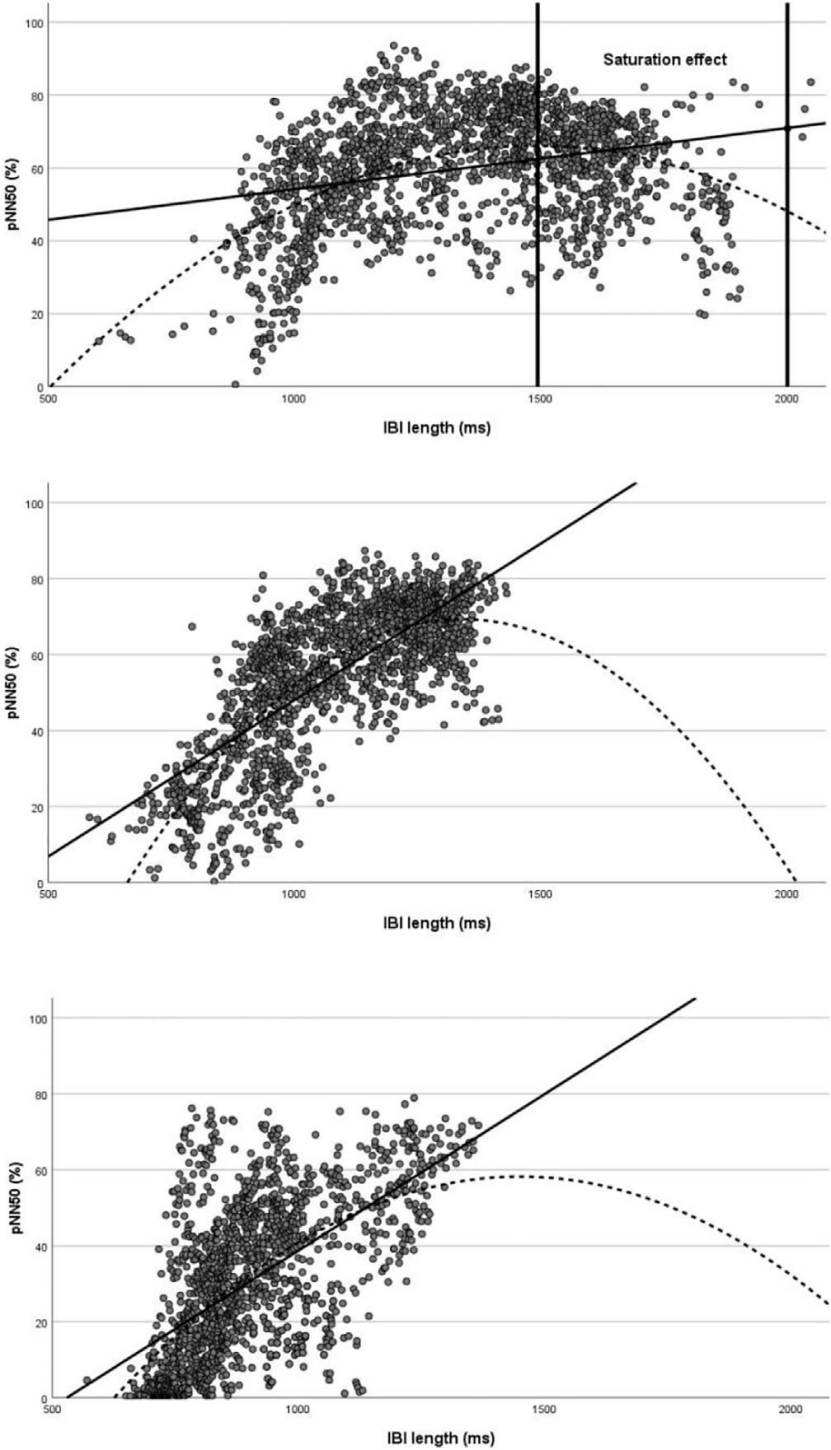
Relationship between pNN50 and interbeat interval (IBI) in patients with anorexia nervosa (AN) at admission (top) and during refeeding treatment (middle) and controls (bottom). The dotted curve represents a quadratic function, whereas the straight line represents a linear function. Patients with AN at admission (quadratic function: pNN50 = −0.000068 × IBI2 + 0.203 × IBI − 85.101 *R* ^2^ = 0.21; linear function: pNN50 = 0.017 × IBI + 37.387 *R* ^2^ = 0.08). Patients with AN at release (quadratic function: pNN50 = 0.403 × IBI − 0.000150 × IBI2–200.35 *R* ^2^ = 0.60; linear function: pNN50 = 0.082 × IBI − 34.356 *R* ^2^ = 0.08). Controls (quadratic function: pNN50 = 0.249 × IBI x 2–0.00008582 × IBI − 122.074 *R* ^2^ = 0.36; linear function: pNN50 = 0.082 × IBI − 43.679 *R* ^2^ = 0.35)

Statistical analysis was performed using SPSS version 25.0. The Shapiro‐Wilk test was used to test whether the samples demonstrated a normal distribution. Therefore, the test showed that the data distribution was partly non‐Gaussian; thus, nonparametric tests were used. First, we compared IBIs and HRV parameters before and after nutritional refeeding using Wilcoxon signed‐rank tests. Second, we compared IBI and HRV values of patients with AN with HRV and IBI values from controls using Mann‐Whitney *U* tests. A *P*‐value of <.01 was considered statistically significant. Third, we examined the relationship between LnHF power and IBI using Spearman's rank correlation coefficient for each control, each patient with AN, and each patient with the *HCN4* c.1737+1G>T mutation. If Spearman's rank correlation coefficient attained a negative value, the relationship between IBI and LnHF power was defined as saturated.^33^ Fourth, the relationship between IBI and pNN50 in patients and controls was plotted and fitted to quadratic and linear models (Figure 2).^33^

## 3 RESULTS

Twenty patients with AN (18 females) were enrolled with a mean body mass index percentile of 2.59 (SD: 6.17) at admission and of 5.92 (SD: 11.30) at discharge. Table 1 summarizes patients' characteristics. The following are relevant characteristics not presented in Table 1 (Appendix B): six patients had low triiodothyronine levels, three patients had hypermagnesemia, three patients had anemia, one patient had hyponatremia, one patient had leukopenia, and one patient had mild pericardial effusion. Furthermore, one patient had postural orthostatic tachycardia syndrome and tested positive for anti‐α‐adrenergic antibodies. It is also worth noting that the patient with the longest disease duration had the highest HRV parameters and lowest mean HR during the night (Appendix B).

### 3.1 HRV and IBI in patients with AN during starvation and refeeding

Table 1 summarizes HRV parameters and IBIs of patients and controls. Upon admission, the HRV parameters of patients with AN were high and HRs were low compared with those of controls (pNN50: +117.28%; RMSSD: +103.42%; LnHF power: +23.74%; IBI: + 36.31%). These differences were statistically significant at a level of *P* < .01 for HR and all HRV parameters (Table 1).

During refeeding, we observed an increase in HR in 20 patients and varying changes in HRV metrics. In 11 patients, pNN50 and RMSSD increased, whereas in nine patients, pNN50 and RMSSD decreased. LnHF power decreased in eight patients, whereas in 12 patients, LnHF power increased. In contrast, the increase in HR during refeeding was statistically significant (*P* < .01). No significant change in HRV parameters was observed. Patients with AN continued to have significantly lower HR and higher HRV parameters during refeeding treatment than controls (Table 1).

### 3.2 HRV and IBI in patients with *HCN4* mutations

Table 1 shows HRV parameters and IBIs of patients with *HCN4* c.1737+1G>T mutation. HRV and IBI values were high in patients with *HCN4* c.1737+1G>T mutation than in controls (pNN50: +121.45%; RMSSD: +122.23%; LnHF power: +24.78%; IBI: +37.49%).

### 3.3 Relationship between IBI and HRV metrics

We observed a positive correlation between IBI and LnHF power in 19 out of 20 controls (mean Spearman's rank‐order correlation coefficient: 0.52). In contrast, patients with AN either had a weak positive correlation between LnHF power and IBI or a negative Spearman's correlation coefficient at admission (mean Spearman's rank‐order correlation coefficient: 0.05). During refeeding, the correlation between LnHF power and IBI strengthened in many patients, approximating normal values (Table 2). In two of four *HCN4* mutation carriers, we observed a positive correlation between LnHF power and IBI (Spearman's rank‐order correlation coefficients: 0.49 and 0.50). However, in two patients, we observed a negative correlation between LnHF power and IBI (rank‐order correlation coefficients: −0.01 and −0.22).

**TABLE 2:** Spearman's correlation coefficients. Significant differences: bold numbers = *P* < .01

Patient number	Age (years)	HF‐IBI correlation at admission	HF‐IBI correlation during re‐feeding	Control number	Age (years)	HF‐IBI correlation
1	12	**0.307**	**0.719**	1	12	**0.819**
2	13	**0.909**	**0.526**	2	13	–0.248
3	14	**–0.434**	–0.035	3	14	**0.755**
4	14	**–0.509**	**–0.381**	4	14	0.679
5	13	**–0.212**	0.196	5	13	**0.381**
6	16	**–0.606**	**–0.532**	6	16	**0.493**
7	13	0.07	**–0.497**	7	13	**0.785**
8	13	**0.661**	**0.347**	8	13	0.137
9	13	**–0.557**	**0.677**	9	13	**0.473**
10	14	**0.273**	–0.066	10	14	**0.81**
11	12	**0.641**	**0.274**	11	12	**0.669**
12	15	**0.345**	**0.31**	12	15	**0.69**
13	16	–0.246	**0.813**	13	16	**0.79**
14	14	**–0.905**	**0.746**	14	14	0.052
15	16	**0.783**	**0.265**	15	16	**0.685**
16	15	**–0.33**	**0.515**	16	15	**0.383**
17	16	0.176	–0.24	17	15	**0.775**
18	15	**0.325**	**–0.336**	18	15	**0.364**
19	13	0.196	**0.888**	19	13	**0.918**
20	13	**0.044**	**0.809**	20	14	**0.600**
Negative Spearman's correlation coefficient		8	7			1

In patients with AN, at admission, the quadratic function was superior to the linear one in describing the relationship between IBI and pNN50 (Figure 2). Moreover, the deflection point in the quadratic regression model occurred in proximity of the mean IBI (deflection point: 1492.65 ms/66.40%). In controls, quadratic and linear functions were equally fitting (Figure 2). The deflection point of the quadratic regression model occurred near to the maximal IBI (deflection point: 1450.71 ms/58.24%; maximal IBI: 1364.82 ms). In the 27‐year‐old patient with *HCN4* c.1737+1G>T mutation, the quadratic function was superior to the linear one in describing the relationship between IBI and HRV metrics (Appendix A), whereas quadratic and linear functions were equally fitting in the 22‐ and 23‐year‐old patients with positive correlation between LnHF power and IBI (Appendix A).

## 4 DISCUSSION

This investigation demonstrated that the relationship between IBI and HRV showed signs of saturation in patients with AN and in two patients with the *HCN4* c.1737+1G>T mutation. In contrast, the signs of HRV saturation were barely present in controls. Additionally, a significant increase in HR during nutritional refeeding was observed, whereas HRV parameters either increased or decreased.

### 4.1 HRV and HR changes during starvation and refeeding

Our findings are highly consistent with studies measuring HRV before nutritional refeeding in patients with acute AN.^34^, ^35^ Earlier studies found increased HRV values in patients with AN.^5^, ^34^, ^35^

After high‐calorie refeeding, the mean IBI values of controls were approximated, whereas HRV parameters either increased or decreased (Table 1). The significant increase in HR during refeeding treatment suggests that bradycardia is an adaptive response to malnutrition in patients with AN.^35^ Bradycardia presumably decreases energy consumption and is effective in regulating emotional stress.^36^

### 4.2 Relationship between IBI and HRV in controls and patients with AN

Recent studies suggest that HRV is partly a nonlinear surrogate for HR.^37^, ^38^, ^39^ Therefore, it was asserted that HRV parameters fundamentally increase with a decrease in HR and an increase in IBI for mathematical and physiological reasons.^16^, ^40^, ^41^, ^42^ Boyett et al concluded that high HRs with steep pacemaker potentials produce relatively (little variability between successive heartbeats) small changes in IBI. In contrast, low HRs with flat pacemaker potentials produce relatively large changes in IBI.^16^ Consequently, HRV parameters were corrected for the chronotropic state.^41^, ^43^, ^44^ Here, we demonstrate that HRV is not simply a surrogate for HR or IBI in patients with AN. We observed dissociation between HRV parameters and IBI in patients with AN (Appendix A and Table 2).

We calculated Spearman's correlation coefficients for the IBI‐LnHF power relationship for each patient and control subject. The fd parameter of LnHF power has been shown to quantify the magnitude of respiratory sinus arrhythmia.^45^ The correlation between IBI and LnHF power was much weaker in patients with AN than in controls. In eight of the 20 patients with AN, we even found a negative Spearman's correlation coefficient. A Spearman's correlation coefficient less than zero implies that most LnHF power values and corresponding IBIs are likely to be located at the descending limb of a quadratic HRV‐IBI function. Consequently, HRV saturation was present in at least eight patients with AN at admission and at least seven patients with AN during refeeding treatment (Table 2).

Spearman's correlation coefficients were similar to those in controls in some patients with AN may be due to the differences in individual IBI‐LnHF power relationships. Moreover, HRV saturation may only occur in patients and controls if a specific IBI is exceeded.

Consequently, the period in which distinct HRV saturation is detectable may be restricted to severe starvation phases, which some patients may not have experienced. Evidence of this may be that signs of HRV saturation partly disappeared during refeeding treatment (Table 2). During nutritional refeeding, Spearman's correlation coefficients increased in 11 of the 20 patients, implying a shift toward the ascending limb of the curve (Table 2).

### 4.3 Relationship between IBI and HRV in patients with *HCN4* mutations

In patients with the *HCN4* c.1737+1G>T mutation, our HRV and IBI analysis revealed contradictory results. In two patients, a strong correlation between IBI and LnHF power was present, which confirmed the theory of HRV boost in *HCN4* mutation carriers.^25^

Two patients had a negative Spearman's correlation coefficient, which implied HRV saturation. In addition, the pNN50/IBI graph indicates HRV saturation in these patients (Appendix A). Possible explanations for the contradictory results are diverse and comprise differences in individual IBI‐HRV relationships or exercise‐induced high parasympathetic tone in patients with signs of HRV saturation. It is, however, likely that the 23‐ and 22‐year‐old patients who showed a strong correlation between IBI and HF power were not sufficiently bradycardic in their sleep to show HRV saturation. In Appendix A, the 23‐ and 22‐year‐old patients did not reach HRs of less than 45 beats per minute.

### 4.4 Neural or nonneural mechanisms?

We revealed that HRV saturation might not result from sustained parasympathetic control of the sinus node, as stated by Goldberger et al.^23^, ^46^ HRV saturation also occurred in *HCN4* mutation carriers; thus, we assume that HRV saturation is a phenomenon that becomes visible in all subjects with strong bradycardia. The level of bradycardia that is necessary for HRV saturation may be subject specific. Our observation of similar HRV dynamics between *HCN4* mutation carriers and patients with AN supports the theory of Boyett et al, who suggested that nonneural mechanisms, such as HCN4 downregulation, are responsible for bradycardia in athletes.^17^

In patients with the *HCN4* c.1737+1G>T mutation, bradycardia is likely attributable to the consequences of the mutation and not to a high parasympathetic tone. The *HCN4* c.1737+1G>T mutation supposedly leads to conformational changes in the C‐linker part of the HCN4 protein, very close to the cyclic adenosine monophosphate (cAMP)‐binding domain. Therefore, the mutation likely affects cAMP binding. The *HCN4* c.1737+1G>T mutation possibly makes HCN4 less sensitive to cAMP, which leads to a decrease in the steepness of diastolic depolarization and creates an imbalance between I_f_ current and the counteracting parasympathetically activated I_KACh_ current.^47^ Reduced cAMP sensitivity and low cAMP concentrations cause a polarizing shift in the voltage dependence of the I_f_ activation curve, meaning that less I_f_ current is available at diastolic potentials.^48^

Consequently, diastolic depolarization time is prolonged, and HR slows down. However, this effect cannot explain HRV saturation. One possible theory is that if cAMP insensitivity and low cAMP concentrations drastically prolong diastolic depolarization via I_f_, other rhythmic intrinsic pacemaker mechanisms may be less influenced by autonomic tone than the funny channel, become dominant, and intervene to prevent sinus arrest.^49^, ^50^ This would explain the phenomenon of low HRV at long RR intervals.

### 4.5 What is new?

Contrary to Boyett et al, we showed that HRV is not a surrogate of HR. In contrast to Boyett et al, we examined patients who partly reached HRs well below 60 beats per minute. In these patients diagnosed with AN or *HCN4* mutations, the linear relationship between HRV and IBI was lost. Moreover, our results indicate that common explanations of HRV saturation may be fundamentally flawed. Therefore, HRV saturation may not prove parasympathetically induced bradycardia. We further showed that neural mechanisms could not be distinguished from nonneural mechanisms using HRV alone. Consequently, we agree with Boyett et al and conclude that HRV analysis may inadequately measure cardiac vagal tone, at least when intrinsic mechanisms are not considered.

### 4.6 Clinical use of HRV and HR monitoring

We demonstrated that during refeeding therapy, HR increased in 20 patients. Therefore, suspected starvation in patients with AN can be confirmed by a decrease in nighttime HR. Additionally, HR monitoring could be used to maximize the likelihood of detecting relapses in an ambulant setting and facilitate timely intervention to avoid severe somatic consequences. In contrast to weight control, nighttime HRs are hard to manipulate and represent a better parameter to assess treatment success, particularly in the absence of standardized weight control. We further noticed that the patient with the longest on‐time duration of AN had the highest LnHF value despite mild anemia. In addition, missing data prevented a thorough statistical analysis. It can be speculated that high HRV parameters are associated with a long on‐time duration of AN and present an ominous sign.

The present investigation suggests that HRV carries other information and not just that related to HR. Due to the saturation effect, nighttime HRV monitoring may be inferior to HR monitoring to assess treatment success. Further, HRV correction formulas should be used with caution in patients with bradycardia. Subject‐specific correction formulas may perform better than standard formulas.^51^

### 4.7 Limitations

The sample size of this investigation was relatively small, particularly the number of patients with *HCN4* mutations. The implication that mechanisms other than autonomic regulation may be responsible for HRV saturation and the associated bradycardia in patients with AN were based on the data of two patients. Moreover, the other two patients showed expected HRV boosts, supporting the theory of increased cardiovagal modulation in patients with AN (Appendix A). In future studies, larger sample sizes are required. Second, we cannot state with certainty whether the *HCN4* c.1737+1G>T mutation has similar effects as HCN4 downregulation. Third, undetected or unconsidered comorbidities may have influenced our results. Because patients with AN often carry several comorbidities, we believe that the resulting HRV and HR values are affected by various comorbid syndromes. Results from several earlier studies confirm the hypothesis that depression can lower HRV and that autonomic dysfunction can contribute to the development of depression.^52^, ^53^, ^54^ Additionally, antidepressants and antipsychotic drugs can lower HRV.^55^ However, in this study, patients with major comorbidities were excluded (see section 2), antidepressants were prescribed with caution, and selective serotonin reuptake inhibitors were preferred.^56^, ^57^ Fourth, confounders such as respiration, daytime exercise, blood pressure fluctuation, and sleep stage were not controlled. Furthermore, patients with AN were not tested for genetic conditions that could have caused bradycardia. Fifth, we only analyzed two Holter ECGs of each patient, one before and one during refeeding. For future studies examining treatment effects, we recommend calculating 2‐ or 3‐day HRV average values. In addition, the administration of omega‐3 fatty acids and other nutritional supplements may have influenced HRV and HR. Sixth, possible differences in intrinsic HR among subjects could have been responsible for the lack of saturation in some subjects. To test if a saturation effect exists in controls at longer IBIs, channel blockers, such as ivabradine, could be used in future studies. Finally, electrolyte abnormalities, undetected arrhythmias, and altered hormone levels could have been responsible for changes in HRV and HR. We assumed that the HRV‐IBI relationship is quadratic with an ascending and descending limb. However, it is likely that the HRV‐IBI relationship is different between individuals and cannot be described using a quadratic function for every subject. Goodness‐of‐fit metrics, such as *R* ^2^, varied to a large extent between patients. In some patients, very low *R* ^2^ values were observed (Appendix A).

## CONCLUSIONS

Our hypothesis, which states that neural mechanisms cause bradycardia in patients with AN, could not be verified. HRV saturation occurs in patients with AN; however, the signs of HRV saturation were also present in *HCN4* mutation carriers. Therefore, the theory of increased cardiovagal modulation as a cause of bradycardia in patients with AN may be unsatisfactory. Metabolic rate and excessive exercise may affect HR via HCN4 regulation. Knowledge of the mechanisms of bradycardia and HRV in patients with AN may lead to the development of novel therapies in the future.

## CONFLICT OF INTEREST

The authors declare there is no conflict of interest.

## AUTHOR CONTRIBUTIONS

Conceptualization: Reiner Buchhorn, Christoph Baumann, Christian Willaschek

Data curation: Reiner Buchhorn, Christoph Baumann, Christian Willaschek

Formal Analysis: Christoph Baumann

Funding Acquisition: Reiner Buchhorn, Christoph Baumann

Investigation: Reiner Buchhorn, Christoph Baumann, Christian Willaschek

Methodology: Reiner Buchhorn, Christoph Baumann

Project administration: Reiner Buchhorn, Christian Willaschek

Resources: Reiner Buchhorn, Christian Willaschek

Supervision: Reiner Buchhorn, Christian Willaschek

Validation: Reiner Buchhorn, Christian Willaschek

Writing**—**Original Draft Preparation: Christoph Baumann

Writing**—**Review and Editing: Reiner Buchhorn, Christoph Baumann, Christian Willaschek

All authors have read and approved the final version of the manuscript.

Reiner Buchhorn and Christoph Baumann (corresponding author) had full access to all the data in this study and take complete responsibility for the integrity of the data and the accuracy of the data analysis.

## TRANSPARENCY STATEMENT

The lead author Reiner Buchhorn affirms that this manuscript is an honest, accurate, and transparent account of the study being reported; that no important aspects of the study have been omitted; and that any discrepancies from the study as planned have been explained.

## Supporting information


**Appendix S1**: Supporting Information

## Data Availability

The datasets analyzed in this study are available from Reiner Buchhorn or Christoph Baumann on reasonable request.

## References

[1] Arcelus J , Mitchell AJ , Wales J , Nielsen S . Mortality rates in patients with anorexia nervosa and other eating disorders. A meta‐analysis of 36 studies. Arch Gen Psychiatry. 2011;68(7):724‐731. 10.1001/archgenpsychiatry.2011.74 21727255

[2] Sullivan PF . Mortality in anorexia nervosa. Am J Psychiatry. 1995;152(7):1073‐1074. 10.1176/ajp.152.7.1073 7793446

[3] Papadopoulos FC , Ekbom A , Brandt L , Ekselius L . Excess mortality, causes of death and prognostic factors in anorexia nervosa. Br J Psychiatry. 2018;194(1):10‐17. 10.1192/bjp.bp.108.054742 19118319

[4] Yahalom M , Spitz M , Sandler L , Heno N , Roguin N , Turgeman Y . The significance of bradycardia in anorexia nervosa. Int J Angiol. 2013;22(02):83‐94. 10.1055/s-0033-1334138 24436590 PMC3709923

[5] Mazurak N , Enck P , Muth E , Teufel M , Zipfel S . Heart rate variability as a measure of cardiac autonomic function in anorexia nervosa: a review of the literature. Eur Eat Disord Rev. 2011;19(2):87‐99. 10.1002/erv.1081 25363717

[6] Kollai M , Bonyhay I , Jokkel G , Szonyi L . Cardiac vagal hyperactivity in adolescent anorexia nervosa. Eur Heart J. 1994;15(8):1113‐1118. 10.1093/oxfordjournals.eurheartj.a060636 7988604

[7] Shah SA , Kambur T , Chan C , Herrington DM , Liu K , Shah SJ . Relation of short‐term heart rate variability to incident heart failure from the multi‐ethnic study of atherosclerosis. Am J Cardiol. 2013;112(4):533‐540. 10.1016/j.amjcard.2013.04.018 23683953 PMC3735865

[8] Singh JP , Larson MG , O'Donnell CJ , et al. Association of hyperglycemia with reduced heart rate variability (The Framingham Heart Study). Am J Cardiol. 2000;86(3):309‐312. 10.1016/s0002-9149(00)00920-6 10922439

[9] Liao D , Carnethon M , Evans GW , Cascio WE , Heiss G . Lower heart rate variability is associated with the development of coronary heart disease in individuals with diabetes: the atherosclerosis risk in communities (ARIC) study. Diabetes. 2002;51(12):3524‐3531. 10.2337/diabetes.51.12.3524 12453910

[10] Christensen JH , Toft E , Christensen MS , Schmidt EB . Heart rate variability and plasma lipids in men with and without ischaemic heart disease. Atherosclerosis. 1999;145(1):181‐186. 10.1016/s0021-9150(99)00052-0 10428309

[11] Binici Z , Mouridsen MR , Kober L , Sajadieh A . Decreased nighttime heart rate variability is associated with increased stroke risk. Stroke. 2011;42(11):3196‐3201. 10.1161/STROKEAHA.110.607697 21921280

[12] da Silva VP , de Oliveira NA , Silveira H , Mello RGT , Deslandes AC . Heart rate variability indexes as a marker of chronic adaptation in athletes: a systematic review. Ann Noninvasive Electrocardiol. 2015;20:108‐118. 10.1111/anec.12237 25424360 PMC6931675

[13] Deus L , Sousa CV , Rosa TS , et al. Heart rate variability in middle‐aged sprint and endurance athletes. Physiol Behav. 2019;205:39‐43. 10.1016/j.physbeh.2018.10.018 30389479

[14] Shu‐Ling Lin SW , Huang C‐Y , Shau‐Ping Shiu SW , et al. Effects of yoga on stress, stress adaption, and heart rate variability among mental health professionals—a randomized controlled trial. Worldviews Evid‐Based Nurs. 2015;12:236‐245. 10.1111/wvn.12097 26220020

[15] Shaffer F , Ginsberg JP . An overview of heart rate variability metrics and norms. Front Public Health. 2017;5:258‐258. 10.3389/fpubh.2017.00258 29034226 PMC5624990

[16] Boyett M , Wang Y , D'Souza A . CrossTalk opposing view: heart rate variability as a measure of cardiac autonomic responsiveness is fundamentally flawed. J Physiol. 2019;597(10):2599‐2601. 10.1113/JP277501 31006856 PMC6826226

[17] Boyett MR , D'Souza A , Zhang H , Morris GM , Dobrzynski H , Monfredi O . Viewpoint: is the resting bradycardia in athletes the result of remodeling of the sinoatrial node rather than high vagal tone? J Appl Physiol. 2013;114(9):1351‐1355. 10.1152/japplphysiol.01126.2012 23288551

[18] D'Souza A , Bucchi A , Johnsen AB , et al. Exercise training reduces resting heart rate via downregulation of the funny channel HCN4. Nat Commun. 2014;5:3775. 10.1038/ncomms4775 24825544 PMC4024745

[19] D'Souza A , Sharma S , Boyett MR . CrossTalk opposing view: bradycardia in the trained athlete is attributable to a downregulation of a pacemaker channel in the sinus node. J Physiol. 2015;593(8):1749‐1751. 10.1113/jphysiol.2014.284356 25871551 PMC4405729

[20] Leicht AS . Bradycardia: changes in intrinsic rate rather than cardiac autonomic modulation. Clin Auton Res. 2013;23(6):343. 23884700 10.1007/s10286-013-0208-8

[21] Heathers J , Nagata J , Murray S . What's at the heart of anorexia nervosa? Reconsidering the physiology of bradycardia in anorexia Nerovsa. PsyArXiV. 2017. 10.31234/osf.io/eza6y

[22] Boyett MR . Last word on point: counterpoint. J Appl Physiol. 2017;123(3):694. 10.1152/japplphysiol.00542.2017 28947628 PMC5625072

[23] Goldberger JJ , Challapalli S , Tung R , Parker MA , Kadish AH . Relationship of heart rate variability to parasympathetic effect. Circulation. 2001;103(15):1977‐1983. 10.1161/01.cir.103.15.1977 11306527

[24] de Geus EJC , Gianaros PJ , Brindle RC , Jennings JR , Berntson GG . Should heart rate variability be “corrected” for heart rate? Biological, quantitative, and interpretive considerations. Psychophysiology. 2018;56:e13287. 10.1111/psyp.13287 30357862 PMC6378407

[25] Kozasa Y , Nakashima N , Ito M , et al. HCN4 pacemaker channels attenuate the parasympathetic response and stabilize the spontaneous firing of the sinoatrial node. J Physiol. 2018;596(5):809‐825. 10.1113/JP275303 29315578 PMC5830425

[26] American Psychiatric Association Diagnostic and Statistical Manual of Mental Disorders. 5th ed. Washington DC; AMER PSYCHIATRIC ASSN PUB; 2013.

[27] Hategan L , Csányi B , Ördög B , et al. A novel 'splice site' HCN4 gene mutation, c.1737+1 G>T, causes familial bradycardia, reduced heart rate response, impaired chronotropic competence and increased short‐term heart rate variability. Int J Cardiol. 2017;241:364‐372. 10.1016/j.ijcard.2017.04.058 28465117

[28] Bobkowski W , Stefaniak ME , Krauze T , et al. Measures of heart rate variability in 24‐h ECGs depend on age but not gender of healthy children. Front Physiol. 2017;8:311. 10.3389/fphys.2017.00311 28572771 PMC5435822

[29] Task Force of the European Society of Cardiology and the north American Society of Pacing and Electrophysiology . Heart rate variability: standards of measurement, physiological interpretation and clinical use. Circulation. 1996;93(5):1043‐1065. 8598068

[30] Shaffer F , McCraty R , Zerr CL . A healthy heart is not a metronome: an integrative review of the heart's anatomy and heart rate variability. Front Psychol. 2014;5:1040. 10.3389/fpsyg.2014.01040 25324790 PMC4179748

[31] Hayano J , Yuda E . Pitfalls of assessment of autonomic function by heart rate variability. J Physiol Anthropol. 2019;38(1):3. 10.1186/s40101-019-0193-2 30867063 PMC6416928

[32] Tarvainen MP , Niskanen J‐P , Lipponen JA , Ranta‐aho PO , Karjalainen PA . Kubios HRV – heart rate variability analysis software. Comput Methods Programs Biomed. 2014;113(1):210‐220. 10.1016/j.cmpb.2013.07.024 24054542

[33] Kiviniemi AM , Hautala AJ , Seppanen T , Makikallio TH , Huikuri HV , Tulppo MP . Saturation of high‐frequency oscillations of R‐R intervals in healthy subjects and patients after acute myocardial infarction during ambulatory conditions. Am J Physiol Heart Circ Physiol. 2004;287(5):H1921‐H1927. 10.1152/ajpheart.00433.2004 15242837

[34] Petretta M , Bonaduce D , Scalfi L , et al. Heart rate variability as a measure of autonomic nervous system function in anorexia nervosa. Clin Cardiol. 1997;20(3):219‐224. 10.1002/clc.4960200307 9068906 PMC6656153

[35] Galetta F , Franzoni F , Prattichizzo F , Rolla M , Santoro G , Pentimone F . Heart rate variability and left ventricular diastolic function in anorexia nervosa. J Adolesc Health. 2003;32(6):416‐421. 10.1016/s1054-139x(03)00048-x 12782452

[36] Park G , Thayer JF . From the heart to the mind: cardiac vagal tone modulates top‐down and bottom‐up visual perception and attention to emotional stimuli. Front Psychol. 2014;5:278. 10.3389/fpsyg.2014.00278 24817853 PMC4013470

[37] Rocchetti M , Malfatto G , Lombardi F , Zaza A . Role of the input/output relation of sinoatrial myocytes in cholinergic modulation of heart rate variability. J Cardiovasc Electrophysiol. 2000;11(5):522‐530. 10826931 10.1111/j.1540-8167.2000.tb00005.x

[38] Zaza A , Lombardi F . Autonomic indexes based on the analysis of heart rate variability: a view from the sinus node. Cardiovasc Res. 2001;50(3):434‐442. 10.1016/s0008-6363(01)00240-1 11376619

[39] Monfredi O , Lyashkov AE , Johnsen A‐B , et al. Biophysical characterization of the underappreciated and important relationship between heart rate variability and heart rate. Hypertension. 2014;64(6):1334‐1343. 10.1161/HYPERTENSIONAHA.114.03782 25225208 PMC4326239

[40] Boyett MR , Wang Y , Nakao S , et al. Point: exercise training‐induced bradycardia is caused by changes in intrinsic sinus node function. J Appl Physiol. 2014;123(3):684‐685. 10.1152/japplphysiol.00604.2017 PMC5625071 28684593

[41] Sacha J . Why should one normalize heart rate variability with respect to average heart rate. Front Physiol. 2013;4:306. 10.3389/fphys.2013.00306 24155724 PMC3804770

[42] Gąsior JS , Sacha J , Jeleń PJ , Zieliński J , Przybylski J . Heart rate and respiratory rate influence on heart rate variability repeatability: effects of the correction for the prevailing heart rate. Front Physiol. 2016;7:356‐356. 10.3389/fphys.2016.00356 27588006 PMC4988971

[43] van den Berg ME , Rijnbeek PR , Niemeijer MN , et al. Normal values of corrected heart‐rate variability in 10‐second electrocardiograms for all ages. Front Physiol. 2018;9:424. 10.3389/fphys.2018.00424 29755366 PMC5934689

[44] Gąsior JS , Sacha J , Pawłowski M , et al. Normative values for heart rate variability parameters in school‐aged children: simple approach considering differences in average heart rate. Front Physiol. 2018;9:1495. 10.3389/fphys.2018.01495 30405445 PMC6207594

[45] Egizio V , Eddy M , Robinson M , Jennings JR . Efficient and cost‐effective estimation of the influence of respiratory variables on respiratory sinus arrhythmia. Psychophysiology. 2011;48(4):488‐494. 10.1111/j.1469-8986.2010.01086.x 20718933 PMC2990812

[46] Plews DJ , Laursen PB , Stanley J , Kilding AE , Buchheit M . Training adaptation and heart rate variability in elite endurance athletes: opening the door to effective monitoring. Sports Med. 2013;43(9):773‐781. 10.1007/s40279-013-0071-8 23852425

[47] Gordan R , Gwathmey JK , Xie L‐H . Autonomic and endocrine control of cardiovascular function. World J Cardiol. 2015;7(4):204‐214. 10.4330/wjc.v7.i4.204 25914789 PMC4404375

[48] Monfredi O , Maltsev VA , Lakatta EG . Modern concepts concerning the origin of the heartbeat. Phys Ther. 2013;28(2):74‐92. 10.1152/physiol.00054.2012 PMC3768086 23455768

[49] Ardell JL , Randall WC , Pomeroy G , Lawton M , Kim T . Autonomic regulation of subsidiary atrial pacemakers during exercise. J Appl Physiol. 1991;70(3):1175‐1183. 2032983 10.1152/jappl.1991.70.3.1175

[50] Sealy WC , Bache RJ , Seaber AV , Bhattacharga SK . The atrial pacemaking site after surgical exclusion of the sinoatrial node. J Thorac Cardiovasc Surg. 1973;65(6):841‐850. 4704236

[51] Malik M , Farbom P , Batchvarov V , Hnatkova K , Camm AJ . Relation between QT and RR intervals is highly individual among healthy subjects: implications for heart rate correction of the QT interval. Heart. 2002;87(3):220‐228. 10.1136/heart.87.3.220 11847158 PMC1767037

[52] Agelink MW , Boz C , Ullrich H , Andrich J . Relationship between major depression and heart rate variability: clinical consequences and implications for antidepressive treatment. Psychiatry Res. 2002;113(1):139‐149. 10.1016/S0165-1781(02)00225-1 12467953

[53] Huang M , Shah A , Su S , et al. Association of depressive symptoms and heart rate variability in Vietnam war‐era twins: a longitudinal twin difference study. JAMA Psychiat. 2018;75(7):705‐712. 10.1001/jamapsychiatry.2018.0747 PMC6059565 29799951

[54] Licht CMM , de Geus EJC , van Dyck R , Penninx BWJH . Association between anxiety disorders and heart rate variability in The Netherlands Study of Depression and Anxiety (NESDA). Psychosom Med. 2009;71(5):508‐518. 10.1097/PSY.0b013e3181a292a6 19414616

[55] O'Regan C , Kenny RA , Cronin H , Finucane C , Kearney PM . Antidepressants strongly influence the relationship between depression and heart rate variability: findings from The Irish Longitudinal Study on Ageing (TILDA). Psychol Med. 2015;45(3):623‐636. 10.1017/S0033291714001767 25075912 PMC4413849

[56] van Zyl LT , Hasegawa T , Nagata K . Effects of antidepressant treatment on heart rate variability in major depression: a quantitative review. Biopsychosoc Med. 2008;2(1):12. 10.1186/1751-0759-2-12 18590531 PMC2478652

[57] Kemp AH , Quintana DS , Gray MA , Felmingham KL , Brown K , Gatt JM . Impact of depression and antidepressant treatment on heart rate variability: a review and meta‐analysis. Biol Psychiatry. 2010;67(11):1067‐1074. 10.1016/j.biopsych.2009.12.012 20138254

