# Supplementary material for: Pathophysiological mechanisms of bradycardia in patients with anorexia nervosa
Source: Health Sci Rep. 2021 Jul 23;4(3):e331. doi: 10.1002/hsr2.331 (PMC8299991; doi:10.1002/hsr2.331)
Supplement: Supplementary file 1 — Appendix S1: Supporting Information [file HSR2-4-e331-s001.pdf]

Appendix A

Relationship between pNN50 and IBI in HCN4 mutation carriers

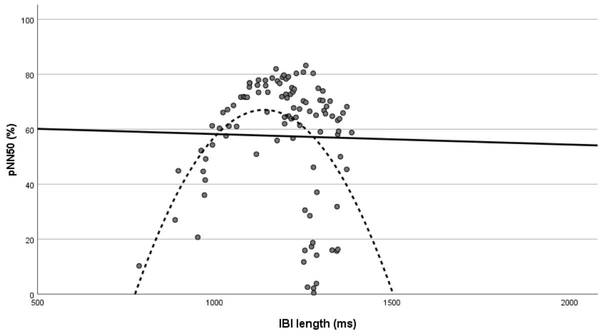

Relationship between pNN50 and IBI in a 27-year-old patient carrying the HCN4 c.1737+1G>T mutation. (Quadratic function: $pNN50 = 1.154 \text{ x } IBI^2 - 0.001 \text{ x } IBI - 589.530$ $R^2 = .197$; linear function: $pNN50 = -0.004 \text{ x } IBI + 62.062$ $R^2 = .000$)

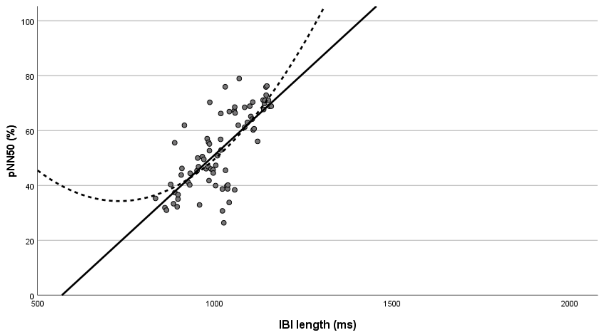

Relationship between pNN50 and IBI in a 23-year-old female patient carrying the HCN4 c.1737+1G>T mutation. (Quadratic function: $pNN50 = -0.309 \text{ x } IBI^2 + 0.000 \text{ x } IBI + 147.054$ $R^2 = .530$; linear function: $pNN50 = 0.119 \text{ x } IBI - 67.501$ $R^2 = 0.517$)

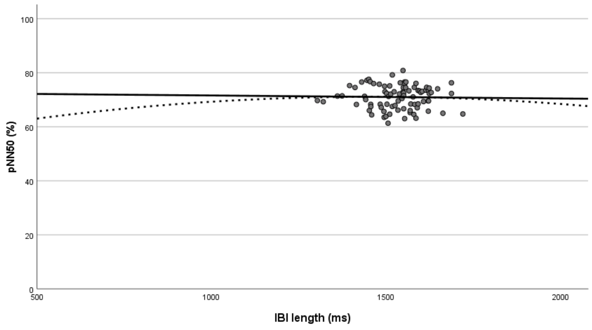

Relationship between pNN50 and IBI in a 13-year-old female patient carrying the HCN4 c.1737+1G>T mutation. (Quadratic function: $\mathrm{pNN50} = -0.026 \times \mathrm{IBI}^2 + 0.000 \times \mathrm{IBI} + 52.324$ $R^2 = .001$; linear function: $\mathrm{pNN50} = -0.001 \times \mathrm{IBI} + 72.698$ $R^2 = .000$)

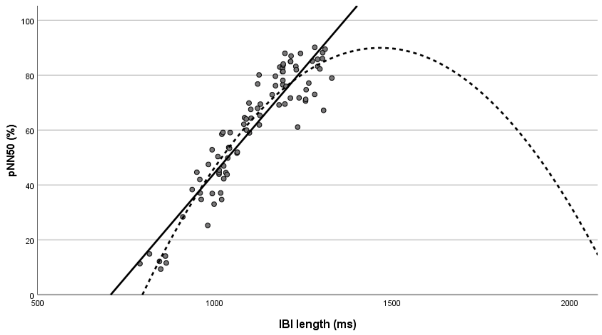

Relationship between pNN50 and IBI in a 22-year-old male patient carrying the HCN4 c.1737+1G>T mutation. pNN50 increases with IBI length. (Quadratic function: $\mathrm{pNN50} = 0.597 \times \mathrm{IBI}^2 - 0.000 \times \mathrm{IBI} - 340.154$ $R^2 = .885$; linear function: $\mathrm{pNN50} = .152 \times \mathrm{IBI} - 106.99$ $R^2 = .853$)

Relationship between pNN50 and IBI in controls

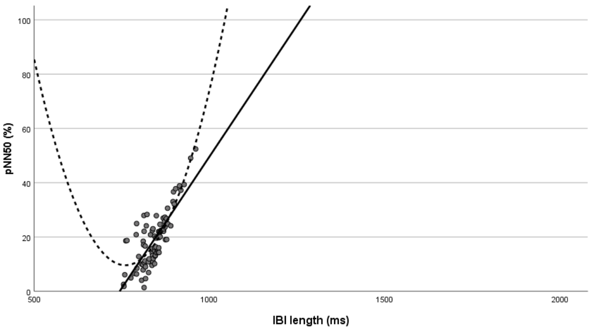

Relationship between pNN50 and IBI in a 12-year old girl. (Quadratic function: $pNN50 = -1.706 \text{ x } IBI^2 - .001 \text{ x } IBI + 657.627$ $R^2 = .702$; linear function: $pNN50 = .194 \text{ x } IBI - 144.160$ $R^2 = .621$)

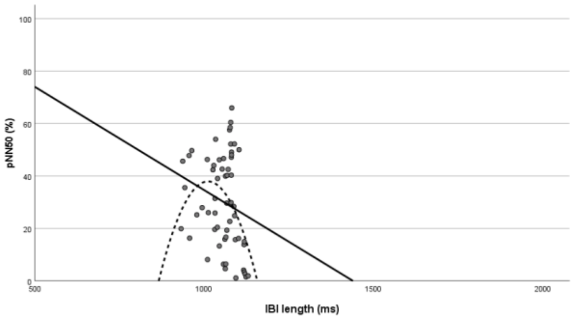

Relationship between pNN50 and IBI in a 13-year old girl. (Quadratic function: $pNN50 = 3.625 \text{ x } IBI^2 - .002 \text{ x } IBI + 657.627$ $R^2 = .127$; linear function: $pNN50 = -.079 \text{ x } IBI - 113.367$ $R^2 = .046$)

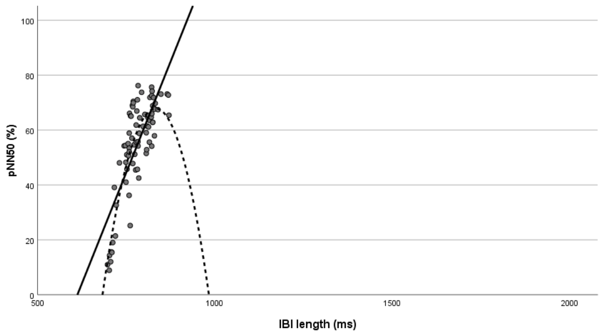

Relationship between pNN50 and IBI in a 14-year old girl. (Quadratic function: pNN50 = 5.029 x IBI^2 − .003 x IBI -2025.828 R^2= .746; linear function: pNN50 = .323 x IBI-197.960 R^2 = .615)

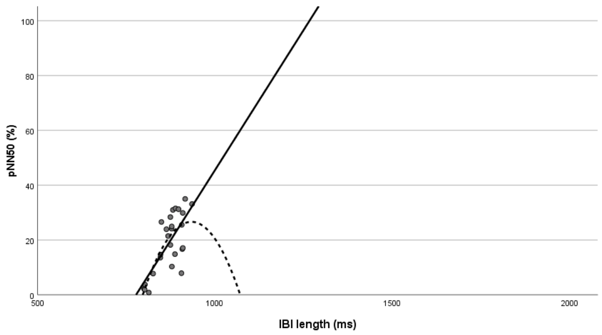

Relationship between pNN50 and IBI in a 14-year old girl. (Quadratic function: pNN50 = 2.639 x IBI^2 − .001 x IBI -159.052 R^2= .568; linear function: pNN50 = .205 x IBI-1204.986 R^2 = .531)

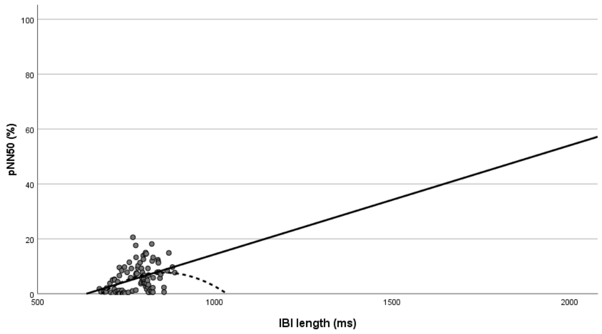

Relationship between pNN50 and IBI in a 13-year old girl. (Quadratic function: pNN50 = .421 x IBI^2 − .000 x IBI -172.433 R^2= .174; linear function: pNN50 = .040 x IBI-25.295 R^2 = .157)

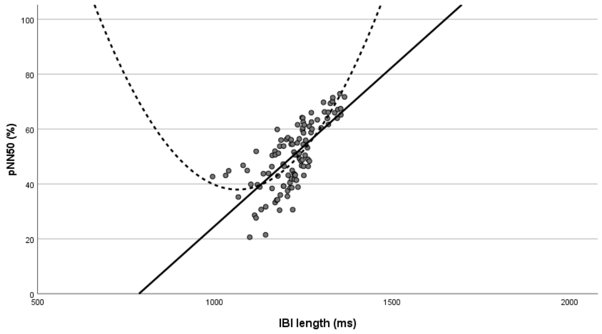

Relationship between pNN50 and IBI in a 16-year old girl. (Quadratic function: pNN50 = -.879 x IBI^2 − .000 x IBI +505.549 R^2= .593; linear function: pNN50 = .116 x IBI-90.938 R^2 = .516)

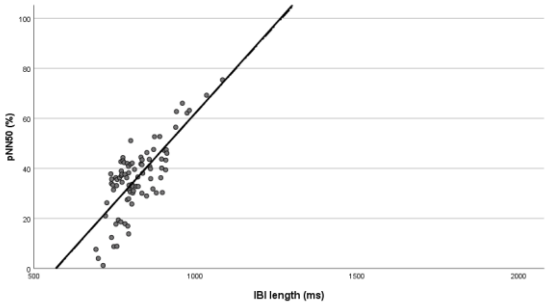

Relationship between pNN50 and IBI in a 16-year old girl. (Quadratic function: pNN50 = .148 x IBI^2 − .000 x IBI -91.578 R^2= .585; linear function: pNN50 = .144 x IBI-81.578 R^2 = .585)

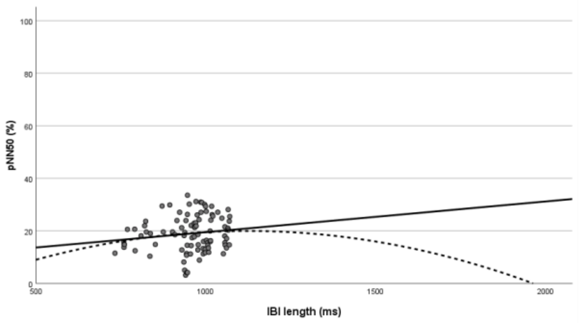

Relationship between pNN50 and IBI in a 13-year old girl. (Quadratic function: pNN50 = .063 x IBI^2 − .000 x IBI -15.670 R^2= .022; linear function: pNN50 = .012 x IBI-7.798 R^2 = .021)

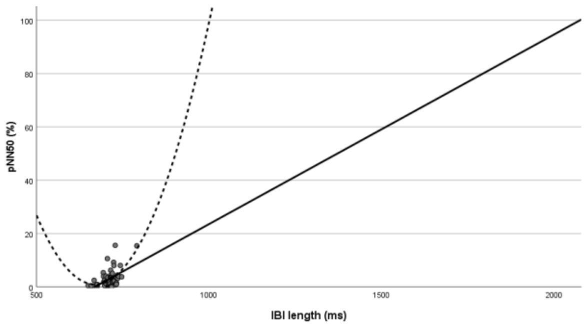

Relationship between pNN50 and IBI in a 13-year old girl. (Quadratic function: pNN50 = -1.207 x IBI^2 − .001 x IBI -404.884 R^2= .332 linear function: pNN50 = .071 x IBI-47.567 R^2 = .248)

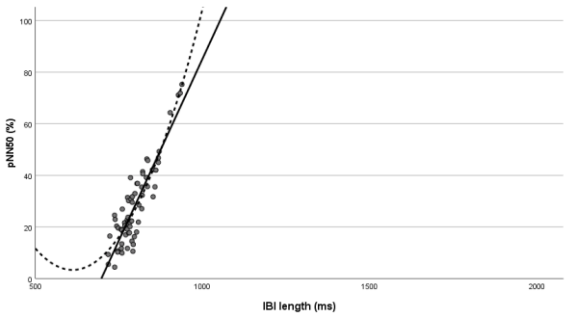

Relationship between pNN50 and IBI in a 14-year old girl. (Quadratic function: pNN50 = -.821 x IBI^2 − .001 x IBI -254.187 R^2= .823 linear function: pNN50 = .282 x IBI-196.394 R^2 = .803)

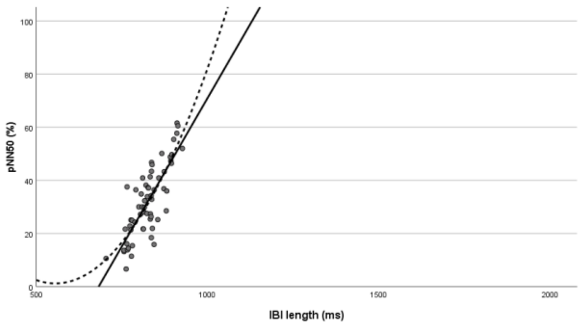

Relationship between pNN50 and IBI in a 12-year old girl. (Quadratic function: pNN50 = -.456 x IBI^2 − .000 x IBI -152. R^2= .659 linear function: pNN50 = .223 x IBI-152.376 R^2 = .651)

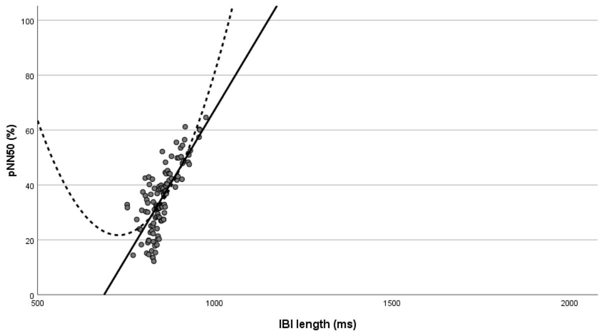

Relationship between pNN50 and IBI in a 12-year old girl. (Quadratic function: pNN50 = -1.172 x IBI^2 +.001 x IBI+447.997 R^2= .598 linear function: pNN50 = .216 x IBI-148.558 R^2 = .568)

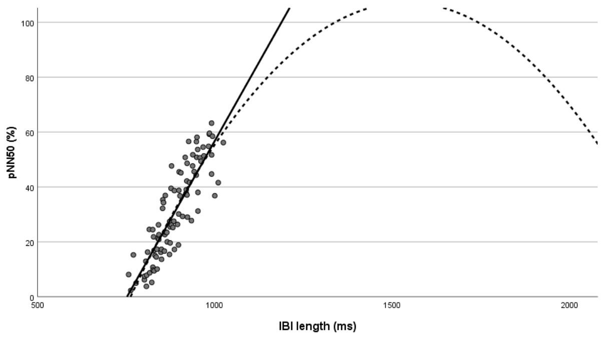

Relationship between pNN50 and IBI in a 12-year old boy. (Quadratic function: pNN50 = .545 x IBI^2 +.000 x IBI-312.713 R^2= .762 linear function: pNN50 = .230 x IBI-172.683 R^2 = .760)

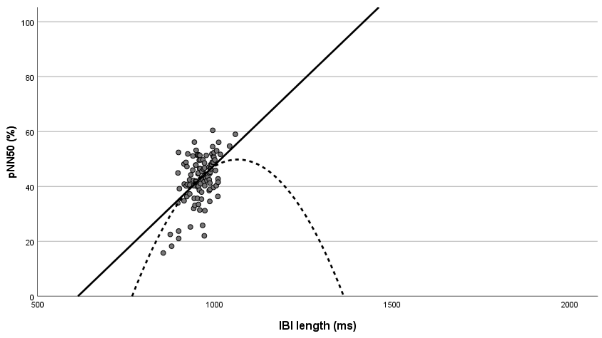

Relationship between pNN50 and IBI in a 14-year old girl. (Quadratic function: pNN50 = 1.193 x IBI^2 -.001 x IBI-584.853 R^2= .271 linear function: pNN50 = .124 x IBI-76.162 R^2 = .257)

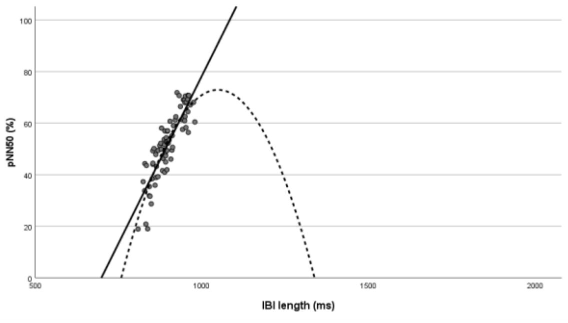

Relationship between pNN50 and IBI in a 16-year old girl. (Quadratic function: pNN50 = 1.819 x IBI^2 -.001 x IBI-879.800 R^2= .772 linear function: pNN50 = .260 x IBI-181.470 R^2 = .756)

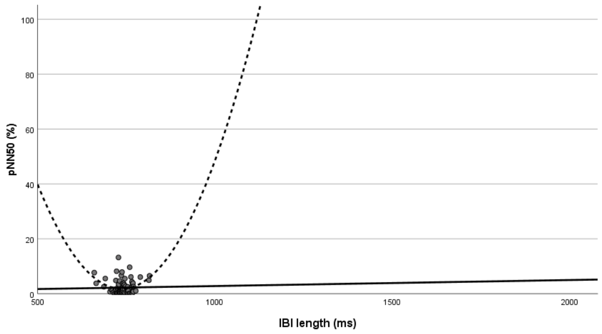

Relationship between pNN50 and IBI in a 14-year old girl. (Quadratic function: pNN50 = -.997 x IBI^2 +.001 x IBI+369.286 R^2= .112 linear function: pNN50 = .002 x IBI+.602 R^2 = .000)

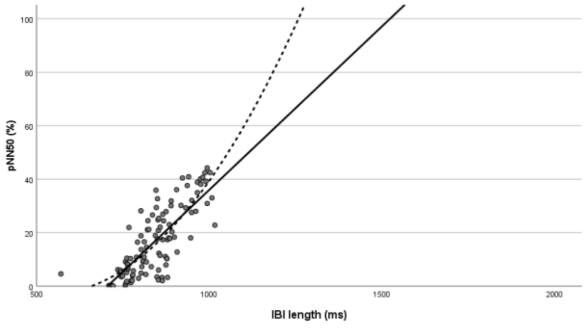

Relationship between pNN50 and IBI in a 15-year old girl. (Quadratic function: pNN50 = -.215 x IBI^2 +.000 x IBI+54.932 R^2= .625 linear function: pNN50 = .122 x IBI-86.314 R^2 = .603)

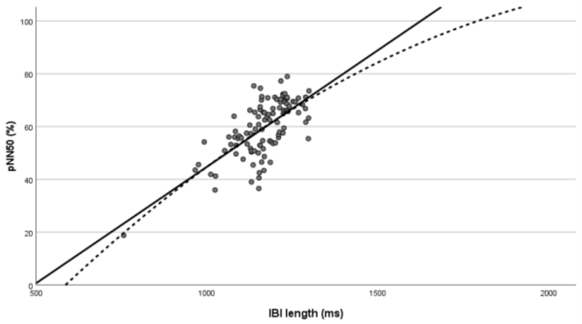

Relationship between pNN50 and IBI in a 15-year old girl. (Quadratic function: pNN50 = .159 x IBI^2 - .000 x IBI-81.972 R^2= .457 linear function: pNN50 = .088 x IBI-43.427 R^2 = .455)

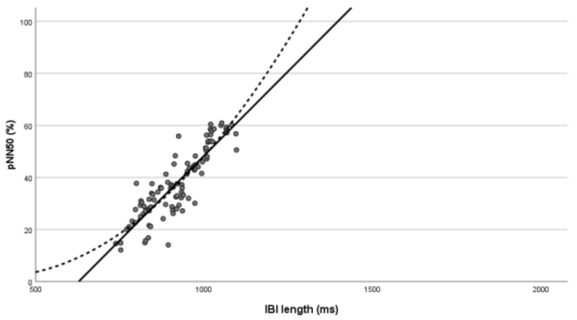

Relationship between pNN50 and IBI in a 13-year old girl. (Quadratic function: pNN50 = -.090 x IBI^2 -.000 x IBI-18.874 R^2= .771 linear function: pNN50 = .130 x IBI-81.664 R^2 = .766)

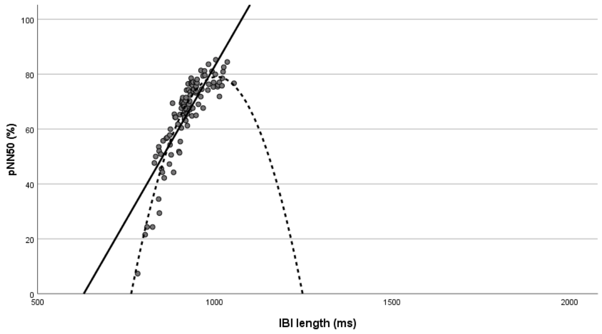

Relationship between pNN50 and IBI in a 15-year old boy. (Quadratic function: pNN50 = 2.723 x IBI^2 -.001 x IBI-1289.238 R^2= .859 linear function: pNN50 = .225 x IBI-141.568 R^2 = .716)

Relationship between pNN50 and IBI in patients with anorexia nervosa at admission and during refeeding

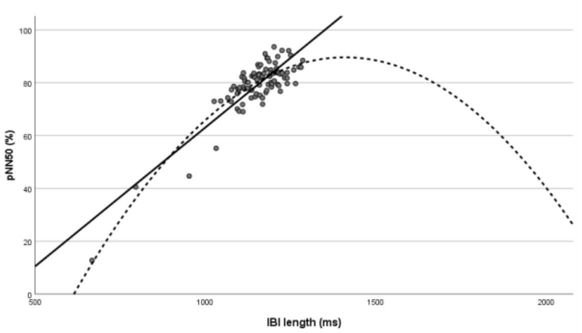

Relationship between pNN50 and IBI in a 12-year old girl with AN at admission. (Quadratic function: $pNN50 = .399 \times IBI^2 + .000 \times IBI - 191.782$ $R^2 = .803$ linear function: $pNN50 = .105 \times IBI - 42.152$ $R^2 = .738$)

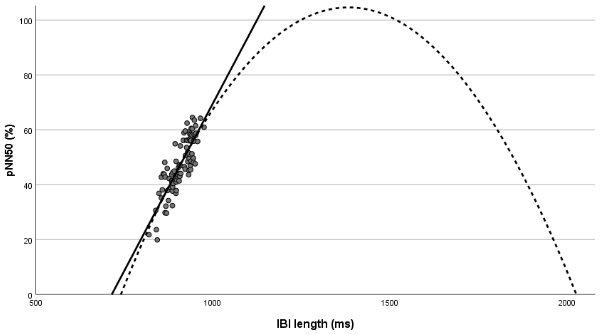

Relationship between pNN50 and IBI in a 12-year old girl with AN during refeeding. (Quadratic function: $pNN50 = .700 \times IBI^2 + .000 \times IBI - 379.343$ $R^2 = .698$ linear function: $pNN50 = .244 \times IBI - 174.259$ $R^2 = .697$)

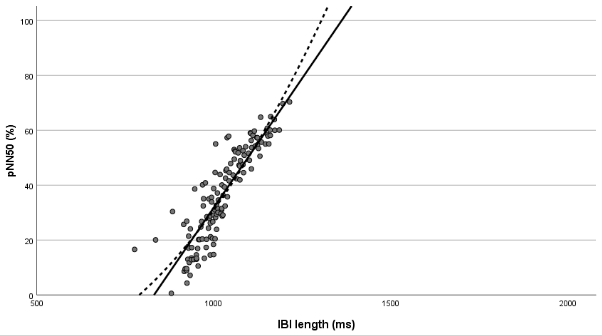

Relationship between pNN50 and IBI in a 13-year old girl with AN at admission. (Quadratic function: pNN50 = -.131 x IBI^2 +.000 x IBI-6.491 R^2= .786 linear function: pNN50 = .189 x IBI-156.788 R^2 = .780)

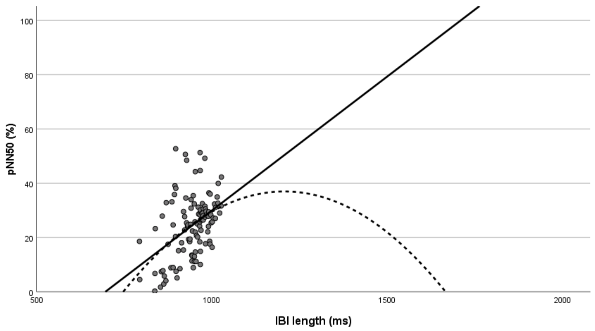

Relationship between pNN50 and IBI in a 13-year old girl with AN during refeeding. (Quadratic function: pNN50 = .422 x IBI^2 +.000 x IBI-217.685 R^2= .195 linear function: pNN50 = .099 x IBI-68.656 R^2 = .193)

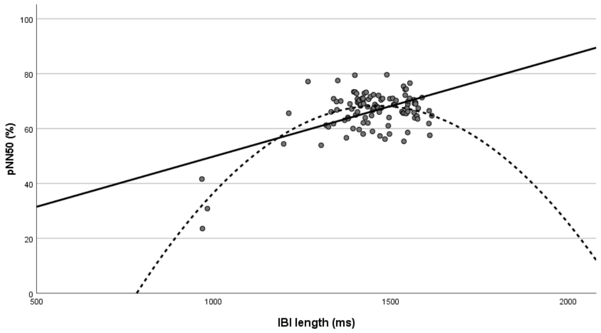

Relationship between pNN50 and IBI in a 14-year old girl with AN at admission. (Quadratic function: pNN50 = .432 x IBI^2 +.000 x IBI-247.699 R^2= .550 linear function: pNN50 = .037 x IBI+13.141 R^2 = .290)

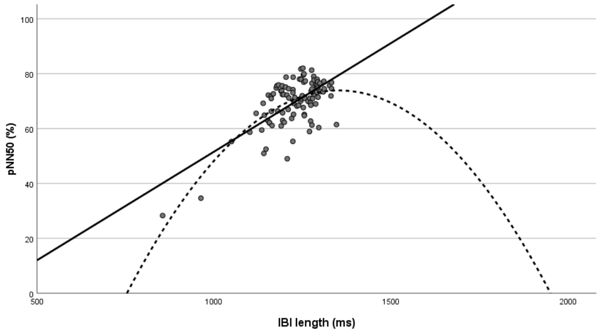

Relationship between pNN50 and IBI in a 14-year old girl with AN during refeeding. (Quadratic function: pNN50 = .559 x IBI^2 +.000 x IBI-303.619 R^2= .515 linear function: pNN50 = .079 x IBI-27.580 R^2 = .436)

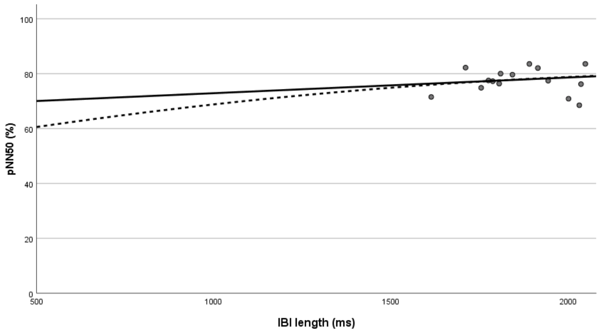

Relationship between pNN50 and IBI in a 14-year old girl with AN at admission. (Quadratic function: pNN50 = .023 x IBI^2 +.000 x IBI+50.159 R^2= .061 linear function: pNN50 = .006 x IBI+67.166 R^2 = .060)

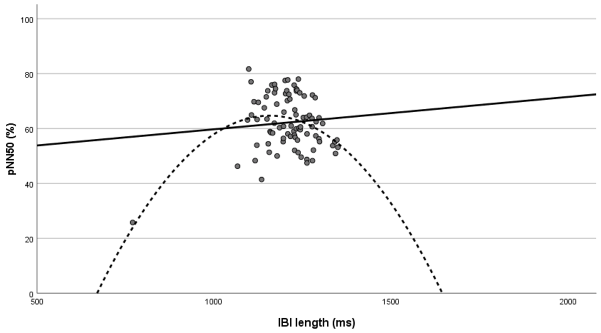

Relationship between pNN50 and IBI in a 14-year old girl with AN during refeeding. (Quadratic function: pNN50 = .630 x IBI^2 +.000 x IBI-299.870 R^2= .235 linear function: pNN50 = .012 x IBI+47.934 R^2 = .009)

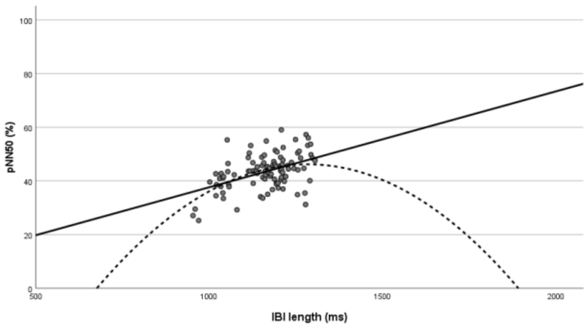

Relationship between pNN50 and IBI in a 13-year old girl with AN at admission. (Quadratic function: pNN50 = .321 x IBI^2 +.000 x IBI-160.022 R^2= .237 linear function: pNN50 = .036 x IBI+1.885 R^2 = .212)

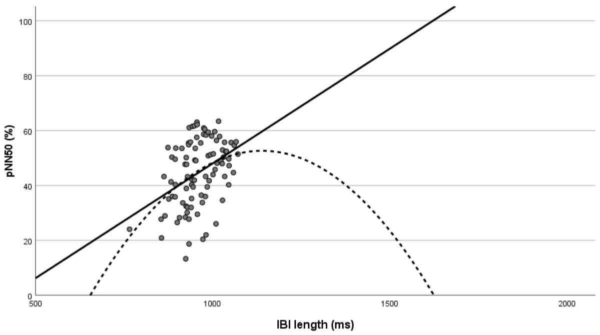

Relationship between pNN50 and IBI in a 13-year old girl with AN during refeeding. (Quadratic function: pNN50 = .510 x IBI^2 +.000 x IBI-237.995 R^2= .168 linear function: pNN50 = .084 x IBI-35.608 R^2 = .160)

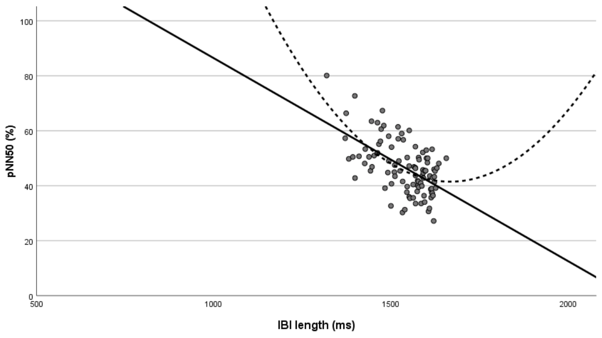

Relationship between pNN50 and IBI in a 16-year old girl with AN at admission. (Quadratic function: pNN50 = -.784 x IBI^2 +.000 x IBI+694.792 R^2= .342 linear function: pNN50 = -.074 x IBI+160.266 R^2 = .321)

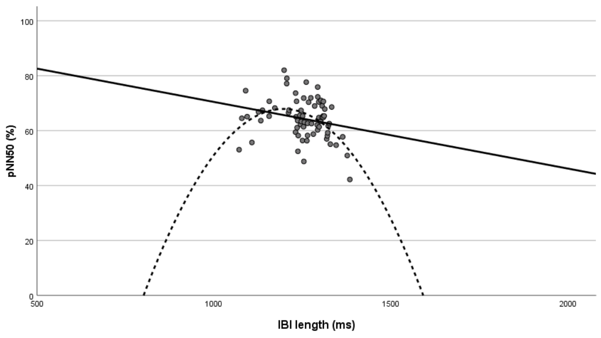

Relationship between pNN50 and IBI in a 16-year old girl with AN during refeeding. (Quadratic function: pNN50 = 1.042 x IBI^2 +.000 x IBI-554.942 R^2= .202 linear function: pNN50 = -.024 x IBI+94.703 R^2 = .056)

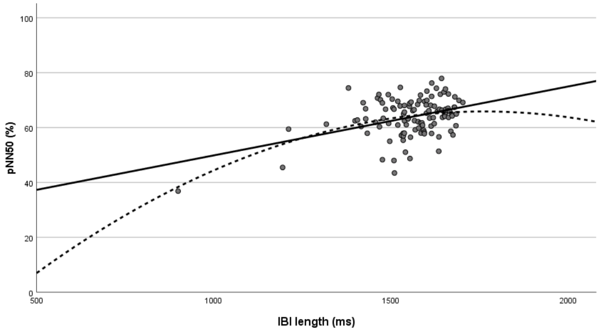

Relationship between pNN50 and IBI in a 13-year old girl with AN at admission. (Quadratic function: pNN50 = .130 x IBI^2 +.000 x IBI-48.984 R^2= .189 linear function: pNN50 = -.025 x IBI+24.744 R^2 = .162)

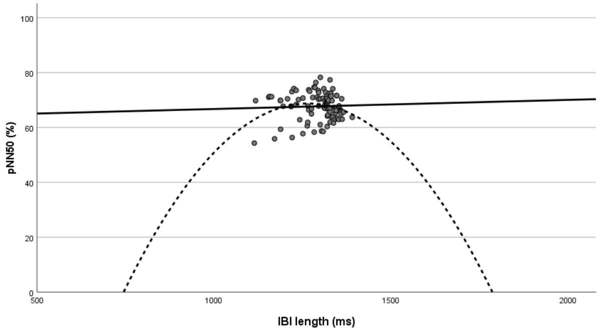

Relationship between pNN50 and IBI in a 13-year old girl with AN during refeeding. (Quadratic function: pNN50 = .642 x IBI^2 +.000 x IBI-337.102 R^2= .046 linear function: pNN50 = -.003 x IBI+63.412 R^2 = .002)

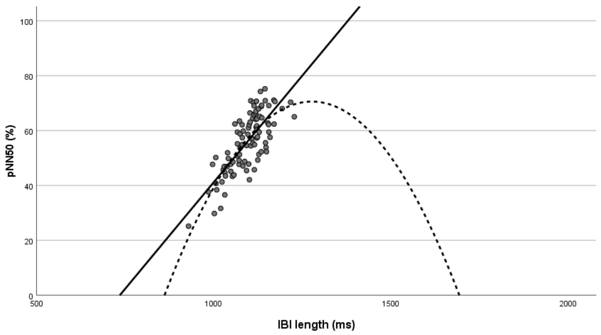

Relationship between pNN50 and IBI in a 13-year old girl with AN at admission. (Quadratic function: pNN50 = 1.042 x IBI^2 +.000 x IBI-594.777 R^2= .608 linear function: pNN50 = .155 x IBI-114.223 R^2 = .583)

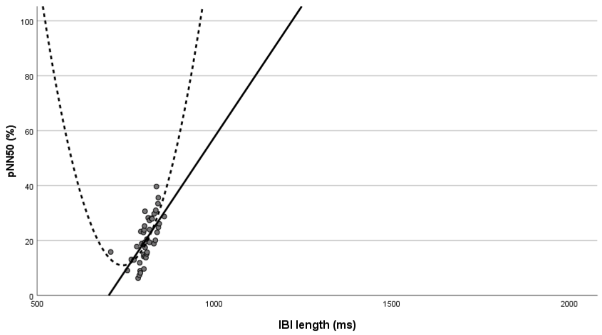

Relationship between pNN50 and IBI in a 13-year old girl with AN during refeeding. (Quadratic function: pNN50 = .642 x IBI^2 +.000 x IBI-337.102 R^2= .046 linear function: pNN50 = -.003 x IBI+63.412 R^2 = .002)

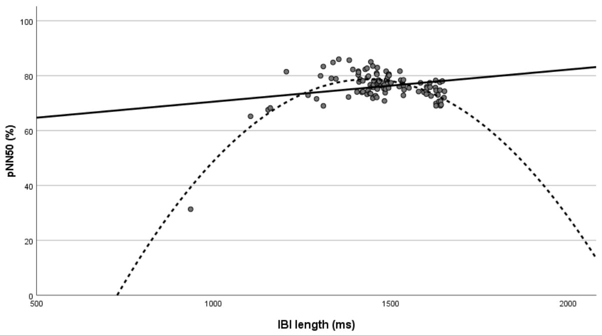

Relationship between pNN50 and IBI in a 13-year old girl with AN at admission. (Quadratic function: pNN50 = .451 x IBI^2 +.000 x IBI-244.991 R^2= .622 linear function: pNN50 = .012 x IBI+58.843 R^2 = .057)

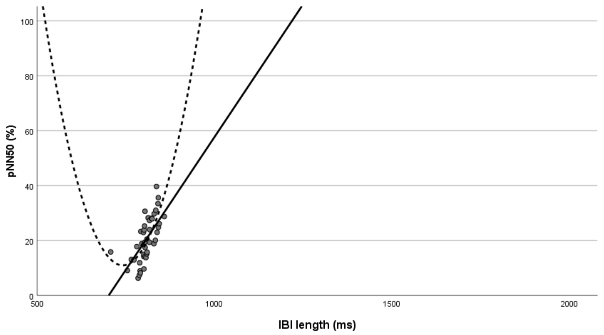

Relationship between pNN50 and IBI in a 13-year old girl with AN during refeeding. (Quadratic function: pNN50 = .642 x IBI^2 +.000 x IBI-337.102 R^2= .046 linear function: pNN50 = -.003 x IBI+63.412 R^2 = .002)

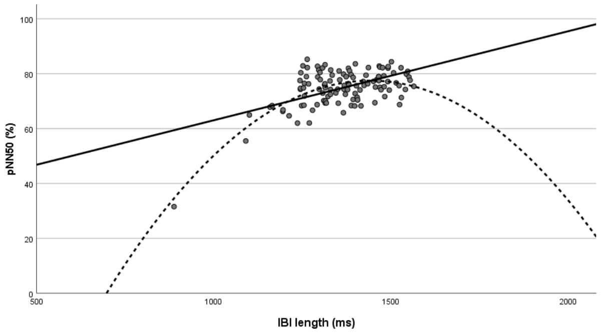

Relationship between pNN50 and IBI in a 14-year old girl with AN at admission. (Quadratic function: pNN50 = .403 x IBI^2 +.000 x IBI-213.252 R^2= .493 linear function: pNN50 = .032 x IBI+30.581 R^2 = .284)

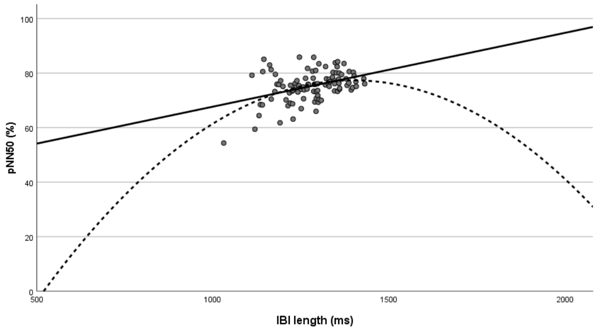

Relationship between pNN50 and IBI in a 14-year old girl with AN during refeeding. (Quadratic function: pNN50 = .280 x IBI^2 +.000 x IBI-118.445 R^2= .182 linear function: pNN50 = .027 x IBI+40.583 R^2 = .159)

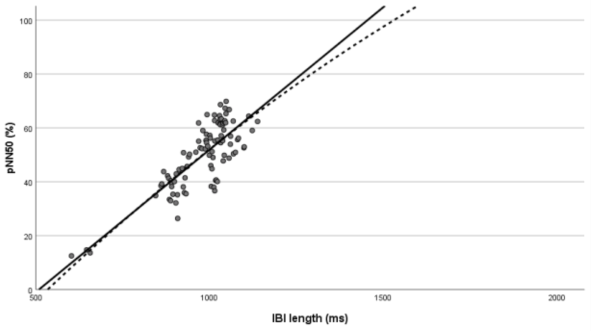

Relationship between pNN50 and IBI in a 12-year old girl with AN at admission. (Quadratic function: pNN50 = .144 x IBI^2 +.000 x IBI-71.059 R^2= .644 linear function: pNN50 = .106 x IBI-53.865 R^2 = .643)

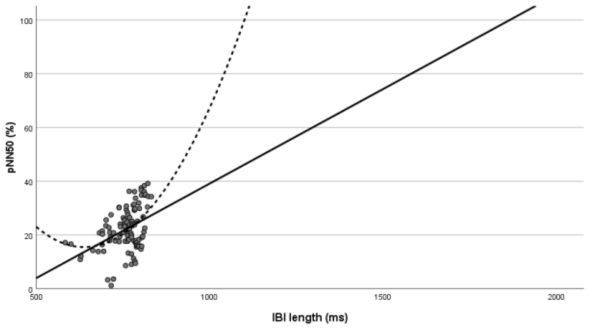

Relationship between pNN50 and IBI in a 12-year old girl with AN during refeeding. (Quadratic function: pNN50 = -.505 x IBI^2 +.000 x IBI+176.566 R^2= .198 linear function: pNN50 = .070 x IBI-31.229 R^2 = .171)

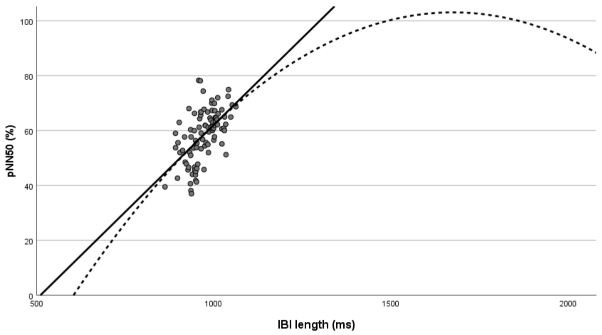

Relationship between pNN50 and IBI in a 15-year old girl with AN at admission. (Quadratic function: pNN50 = .301 x IBI^2 +.000 x IBI-149.181 R^2= .308 linear function: pNN50 = .127 x IBI-64.806 R^2 = .307)

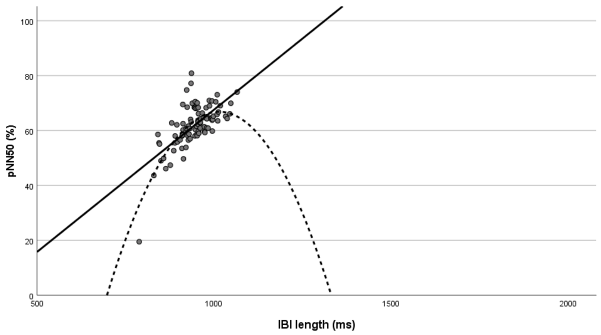

Relationship between pNN50 and IBI in a 15-year old girl with AN during refeeding. (Quadratic function: pNN50 = 1.353 x IBI^2 -.001 x IBI-619.019 R^2= .547 linear function: pNN50 = .104 x IBI-36.078 R^2 = .441)

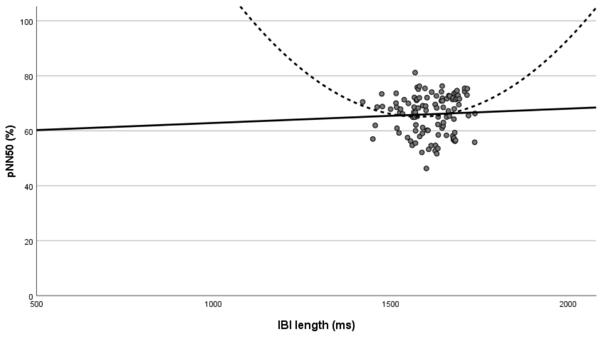

Relationship between pNN50 and IBI in a 16-year old boy with AN at admission. (Quadratic function: pNN50 = -.500 x IBI^2 +.000 x IBI-459.392 R^2= .019 linear function: pNN50 = .005 x IBI+57.631 R^2 = .002)

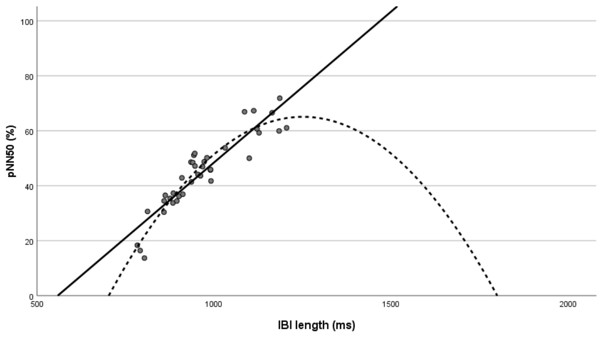

Relationship between pNN50 and IBI in a 16-year old boy with AN during refeeding. (Quadratic function: pNN50 = .541 x IBI^2 +.000 x IBI-273.430 R^2= .889 linear function: pNN50 = .110 x IBI-61.313 R^2 = .840)

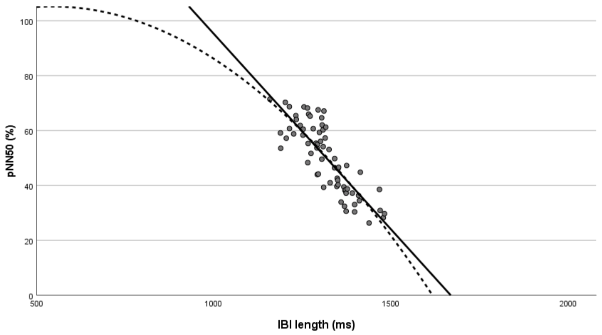

Relationship between pNN50 and IBI in a 14-year old girl with AN at admission. (Quadratic function: pNN50 = .099 x IBI^2 +.000 x IBI+78.331 R^2= .691 linear function: pNN50 = -.143 x IBI+237.726 R^2 = .689)

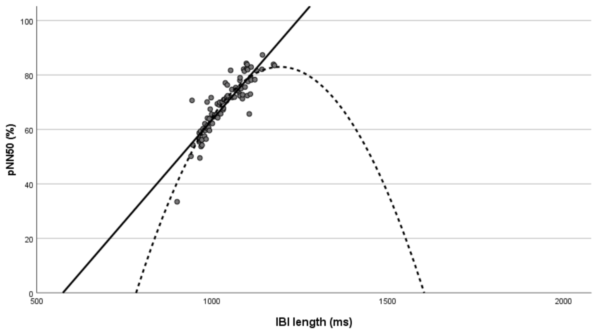

Relationship between pNN50 and IBI in a 14-year old girl with AN during refeeding. (Quadratic function: pNN50 = 1.178 x IBI^2 +.000 x IBI-619.448 R^2= .827 linear function: pNN50 = .150 x IBI-85.944 R^2 = .790)

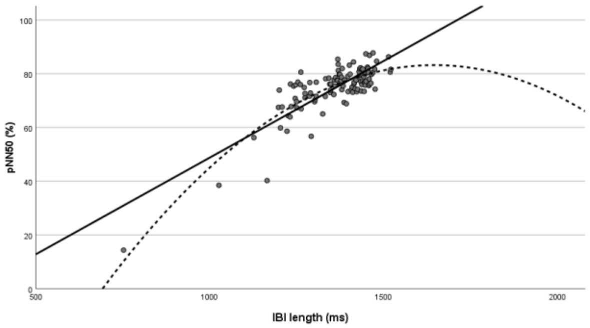

Relationship between pNN50 and IBI in a 16-year old girl with AN at admission. (Quadratic function: $pNN50 = .301 \times IBI^2 - .000 \times IBI - 164.468$ $R^2 = .736$ linear function: $pNN50 = .072 \times IBI - 23.164$ $R^2 = .665$)

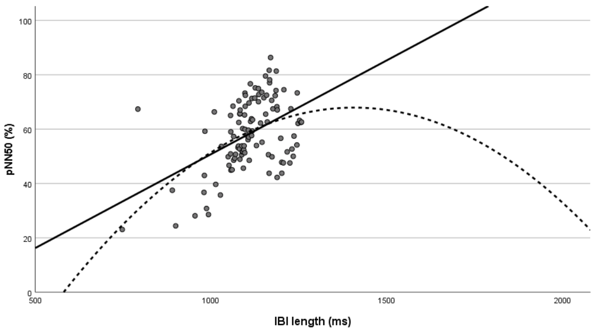

Relationship between pNN50 and IBI in a 16-year old girl with AN during refeeding. (Quadratic function: $pNN50 = .280 \times IBI^2 - .000 \times IBI - 129.086$ $R^2 = .232$ linear function: $pNN50 = .069 \times IBI - 18.304$ $R^2 = .219$)

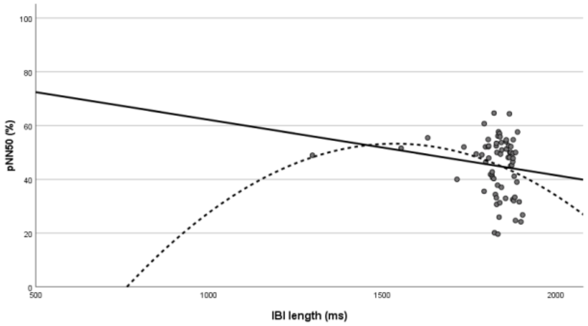

Relationship between pNN50 and IBI in a 16-year old girl with AN at admission. (Quadratic function: pNN50 = .274 x IBI^2 -.000 x IBI-157.102 R^2= .049 linear function: pNN50 = -.021 x IBI-82.746 R^2 = .028)

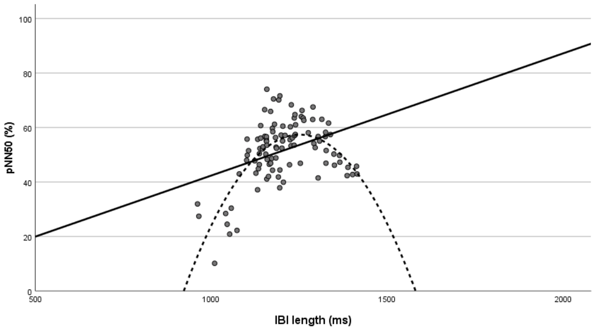

Relationship between pNN50 and IBI in a 15-year old girl with AN during refeeding. (Quadratic function: pNN50 = 1.324 x IBI^2 -.001 x IBI-770.840 R^2= .483 linear function: pNN50 = .045 x IBI-2.490 R^2 = .140)

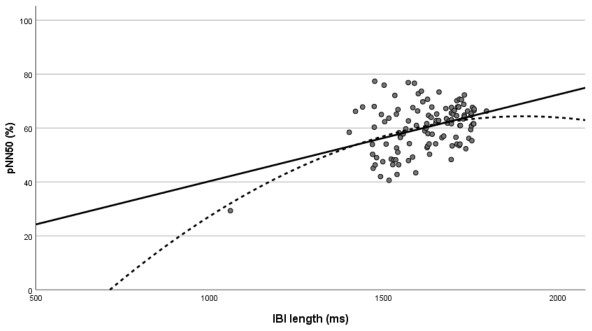

Relationship between pNN50 and IBI in a 16-year old girl with AN at admission. (Quadratic function: pNN50 = .173 x IBI^2 -.000 x IBI-100.176 R^2= .174 linear function: pNN50 = .032 x IBI+8.211 R^2 = .157)

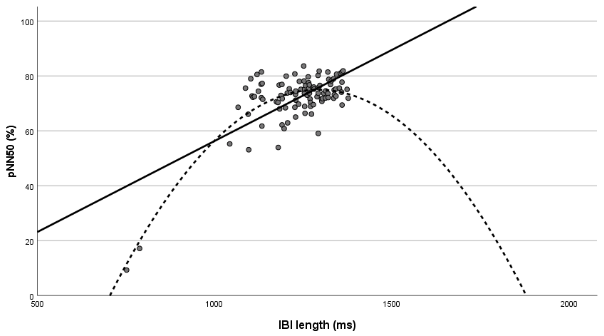

Relationship between pNN50 and IBI in a 16-year old girl with AN during refeeding. (Quadratic function: pNN50 = .562 x IBI^2 +.000 x IBI-288.037 R^2= .689 linear function: pNN50 = .066 x IBI-10.025 R^2 = .454)

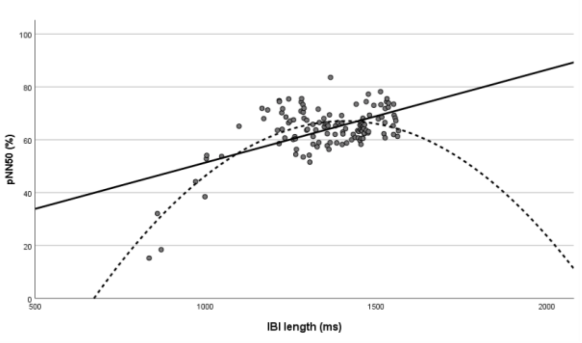

Relationship between pNN50 and IBI in a 15-year old girl with AN at admission. (Quadratic function: pNN50 = .349 x IBI² +.000 x IBI-178.776 R^2= .528 linear function: pNN50 = .035 x IBI+16.306 R^2 = .320)

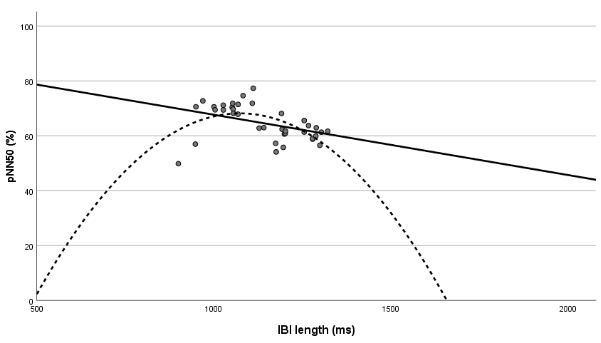

Relationship between pNN50 and IBI in a 15-year old girl with AN during refeeding. (Quadratic function: pNN50 = .430 x IBI² +.000 x IBI-162.444 R^2= .324 linear function: pNN50 = -.022 x IBI+89.633 R^2 = .163)

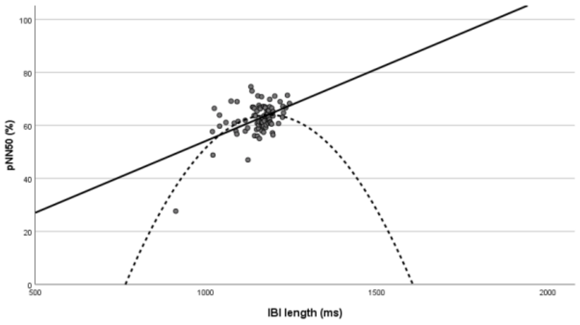

Relationship between pNN50 and IBI in a 13-year old girl with AN at admission. (Quadratic function: pNN50 = .858 x IBI^2 +.000 x IBI-443.914 R^2= .336 linear function: pNN50 = .054 x IBI-.116 R^2 = .235)

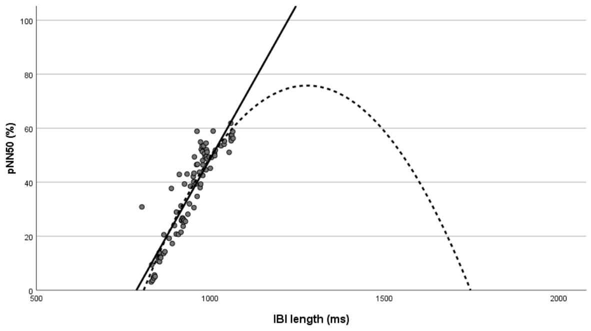

Relationship between pNN50 and IBI in a 15-year old girl with AN during refeeding. (Quadratic function: pNN50 = .880 x IBI^2 +.000 x IBI-485.876 R^2= .843 linear function: pNN50 = .230 x IBI-180.849 R^2 = .823)

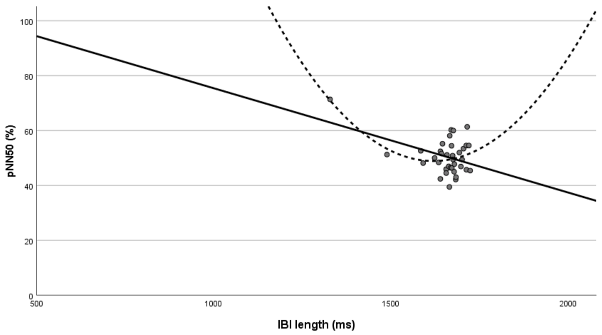

Relationship between pNN50 and IBI in a 13-year old boy with AN at admission. (Quadratic function: pNN50 = -.843 x IBI^2 +.000 x IBI+731.506 R^2= .328 linear function: pNN50 = -.038 x IBI+113.406 R^2 = .181)

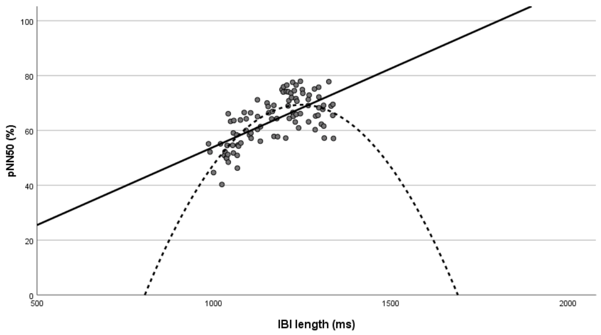

Relationship between pNN50 and IBI in a 13-year old boy with AN during refeeding. (Quadratic function: pNN50 = .885 x IBI^2 +.000 x IBI-481.796 R^2= .570 linear function: pNN50 = .057 x IBI-3.071 R^2 = .440)
